# Transposable elements as evolutionary catalysts of ant macrodiversity

**DOI:** 10.1126/sciadv.aee0306

**Published:** 2026-09-23

**Authors:** Lukas Schrader, Janina L. Rinke, Mohammed Errbii, Scott J. Teresi, Esther van den Bos, Zijun Xiong, Joel Vizueta, Jacobus J. Boomsma, Jürgen Gadau, Guojie Zhang

**Affiliations:** ^1^ Institute for Evolution and Biodiversity, University of Münster, Münster 48149, Germany.; ^2^ Department of Horticulture, Michigan State University, East Lansing, MI 48824, USA.; ^3^ Genetics and Genome Sciences Program, Michigan State University, East Lansing, MI 48824, USA.; ^4^ School of Basic Medical Sciences, Jiangxi Medical College, Nanchang University, Nanchang 330006, China.; ^5^ Section for Ecology and Evolution, Department of Biology, University of Copenhagen, Copenhagen DK-2100, Denmark.; ^6^ Center for Evolutionary and Organismal Biology, Women’s Hospital and Liangzhu Laboratory, Zhejiang University School of Medicine, Hangzhou 310053, China.; ^7^ Key Laboratory of Genetic Evolution and Animal Models, Kunming Institute of Zoology, Chinese Academy of Sciences, Kunming 650023, China.; ^8^ Villum Center for Biodiversity Genomics, Department of Biology, University of Copenhagen, Copenhagen DK-2100, Denmark.

## Abstract

Transposable elements (TEs) are key contributors to genome evolution, yet their macroevolutionary roles in adaptive radiations remain poorly understood, as high-quality genomic resources at sufficient phylogenetic breadth and appropriate comparative methods have been lacking. Here, we analyze 163 genomes spanning 12 of 16 extant ant subfamilies to characterize TE impacts over the past 100 million years. We identify convergent TE acquisition bursts in ancestors of the most speciose ant clades, preceding rapid diversification following the Cretaceous-Paleogene extinction event approximately 66 million years ago. We demonstrate that TEs associate with expansions of odorant receptors and other key gene families and reveal notable compartmentalization of ant genomes into faster-evolving TE-rich and more conserved TE-poor regions. Our findings demonstrate the broad-scale impact of TE-dependent genome evolution during ant adaptive radiation and suggest that similar comparative analyses across other major lineages will be worthwhile to reconstruct the wider macroevolutionary impact of TEs.

## INTRODUCTION

The ants are the largest family of social insects, comprising >15,000 extant species and accounting for a substantial proportion of the terrestrial animal biomass (*1*). Their ecological ubiquity reflects their integral roles in soil aeration, nutrient cycling and other ecosystem functions (*2*). This ecological dominance is underpinned by remarkable phenotypic and behavioral diversity, including substantial variation in colony structure, diet, and caste polymorphism (*3*). The ants’ evolutionary success is rooted in their outstanding social organization, which evolved as a major evolutionary transition to superorganismal colony life (*4*, *5*) over 150 million years ago (Ma) and was instrumental for subsequent adaptive radiation (*6*).

Compared to other animals, ant genomes stand out by their elevated rates of gene turnover and loss of synteny (*7*), features that have in part been linked to lineage-specific adaptations (*8*, *9*). Transposable elements (TEs) offer a credible potential explanation for at least part of this dynamic variation, but no study has yet addressed in any depth the impact of TEs throughout the ants’ evolutionary history.

TEs are generally known to induce genetic and eventually epigenetic variation (*10*–*12*), including effects on gene regulation and genome architecture (*10*, *13*, *14*). They have emerged as key players in the formation of reproductive barriers during species divergence (*13*, *15*) by generating either genetic barriers to gene flow (*16*) or postzygotic hybrid dysgenesis (*17*). Consequently, TEs have contributed to macroevolutionary diversification (*18*–*20*) in birds, fish, bats, primates, and other vertebrates (*21*–*26*), yet no such documentation exists for any major invertebrate clade.

An intriguing characteristic of TEs is their association with elevated evolvability under stress (*10*), suggesting that they may respond to ecological disruption. While numerous studies have linked adaptive radiations to the ecological upheaval following the Cretaceous-Paleogene extinction event ∼66 Ma (*27*–*32*), few have examined the genomic changes accompanying these diversifications (*33*–*35*), and none have assessed whether TE proliferation coincides with this postextinction diversification despite their recognized role in generating genomic innovation.

The ants present an attractive system to address this gap, with more than 15,000 extant species and TEs estimated to comprise 10 to 40% of their genomes (*36*, *37*), making them well suited for exploring the relationship between TE dynamics and adaptive radiation.

Ant TEs have been particularly associated with the expansion of the odorant receptor (OR) gene family through aberrant transposition or recombination (*38*, *39*), yet comprehensive phylogenetic evidence linking TEs with ORs and other gene families remains lacking. Early genomic studies revealed that ants underwent a massive expansion of ORs relative to other Hymenoptera, attributed to the requirements of complex social colony life (*40*, *41*). Gene families associated with cuticular hydrocarbon production, including cytochrome p450s (CyP450), desaturases (*40*), and fatty acid synthases (FAS) (*7*), show similarly expanded copy numbers, reflecting their role in nestmate recognition signaling.

To elucidate the general impact of TEs on genome architecture and evolutionary diversification in ants, we exploited high-quality genome assemblies for 163 ant species representing the phylogenetic and ecological breadth of this insect family (*7*). Using innovative comparative genomic and phylogenomic analyses, we assess the impact of TEs across ant lineages over the last 100 Ma. We demonstrate that genome-wide TE content correlates significantly with species- and genus-level diversification across ant subfamilies and identify convergent TE proliferation bursts in the early Paleogene that preceded independent adaptive radiation within the largest subfamilies. In addition, we show that TE prevalences are associated with expansions of key gene families and are accompanied by notable compartmentalization of genomes into rapidly evolving TE-rich and more conserved TE-poor regions.

## RESULTS

### The repeat landscape of ant genomes

**Fig. 1.**
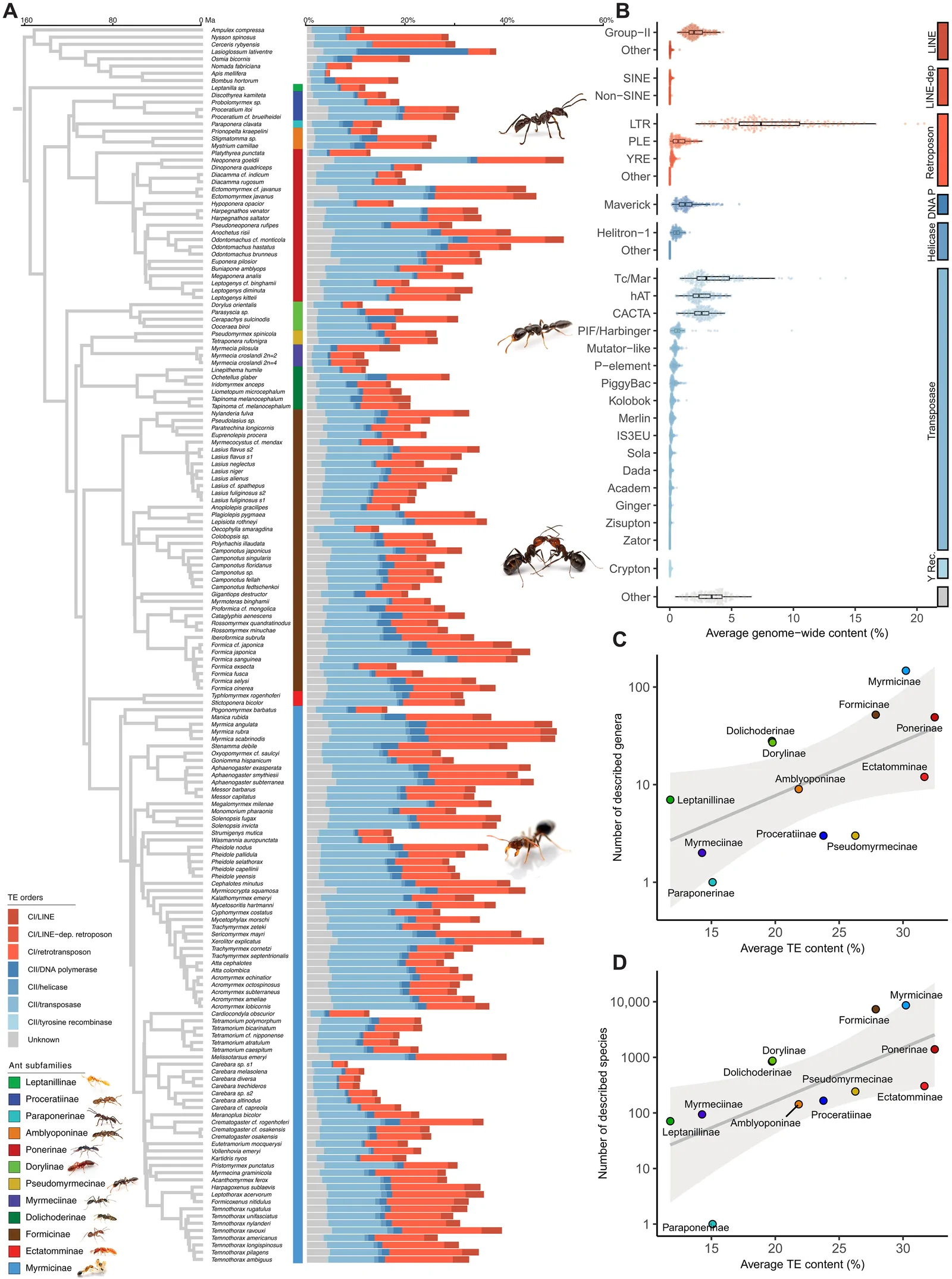
TE prevalence in ant genomes and correlations with lineage diversification. (**A**) Time-calibrated phylogeny (*7*) and annotated TE contents of 163 ant genomes and eight Apoidea genomes. Colored bars next to species names denote different ant subfamilies, and stacked horizontal bar plots show genome-wide proportions of different TE orders. Photographs show representative species of different subfamilies (Photo Credits: A. Wild, Univ. of Texas; C. Liu, Zhejiang University; C. Liao, Chinese Academy of Sciences; L. Schrader; table S2). (**B**) Relative presence of different orders of class I (red) and class II (blue) TEs (*y* axis) partitioned by subclass [colored boxes on the right, see legend in (A)] across the 163 ant genomes. Subclasses from top to bottom: class I (red): long-interspersed nuclear elements (LINE); nonautonomous LINE-dependent retroposons and retrotransposons; class II (blue): polymerase-, helicase-, transposase-, and tyrosine recombinase-based DNA transposons. Unclassified and other low-frequency TEs are pooled as “other” in gray. SINE, short interspersed nuclear elements; PLE, Penelope-like elements; YRE, tyrosine recombinase retroelements. (**C** and **D**) Average genome-wide TE content (*x* axis) in the different ant subfamilies predicts the number of known extant genera (C) and species (D) per subfamily (log_10_-scaled, *y* axes) according to phylogenetic regression analyses. Each dot represents one ant subfamily, colored according to the legend in (A). Linear regressions are derived from *pgls* models. Gray shadings are 95% confidence intervals (CI).

We annotated TEs in the genomes of 163 ants and eight Apoidea outgroup species (*7*), using a joint TE library comprising curated consensus de novo repeat models from these 171 species (with 18% of consensus models predicted to be complete; figs. S1 and S2) and curated models from other arthropods. The genomes included chromosome-level assemblies (*n* = 16 for ants, *n* = 7 for outgroup species), highly contiguous PacBio long-read assemblies (*n* = 114; all ants), and assemblies based on short reads (*n* = 33 for ants; *n* = 1 for outgroups) (*7*). TEs are a major contributor to ant genome size variation (Pearson correlation coefficient *r* = 0.90, *P* < 0.00001; fig. S3), making up between 8% in *Carebara* sp. *s1* (∼17 Mb of the 195 Mb total genome size) and 53% in *Odontomachus* cf. *monticola* (∼318 Mb of the 595 Mb total genome size), with an average TE content of 28% across the ant genomes analyzed (Fig. 1A and table S1).

On average, DNA transposons were slightly more common (13.6% of the average genome-wide content) than RNA transposons (11.5%) (table S1 and fig. S4), while unclassified TEs accounted for ∼3%, which is considerably less than in previously reports estimating these TEs to represent 9 to 13% (*36*, *37*). This difference likely reflects the more conservative curation and filtering of TE libraries in the present study, removing a greater proportion of repeat elements incorrectly classified as TEs. The most common TE superfamilies were long-terminal repeat (LTR) retrotransposons (8.3%) and Tc1/Mariner DNA transposons (3.7%) (Fig. 1B). Overall, average TE content was significantly and positively associated with the number of described genera per subfamily [phylogenetic generalized least squares (*pgls*) model, *P* < 0.043, adj. *R*^2^ = 0. 29; Fig. 1C] and the number of described species per subfamily (*pgls* model, *P* < 0.024, adj. *R*^2^ = 0.36; Fig. 1D), indicating that TE expansions are significantly correlated with evolutionary diversification at the lower taxonomic levels.

### TE proliferations preceded the adaptive radiations of ant subfamilies

**Fig. 2.**
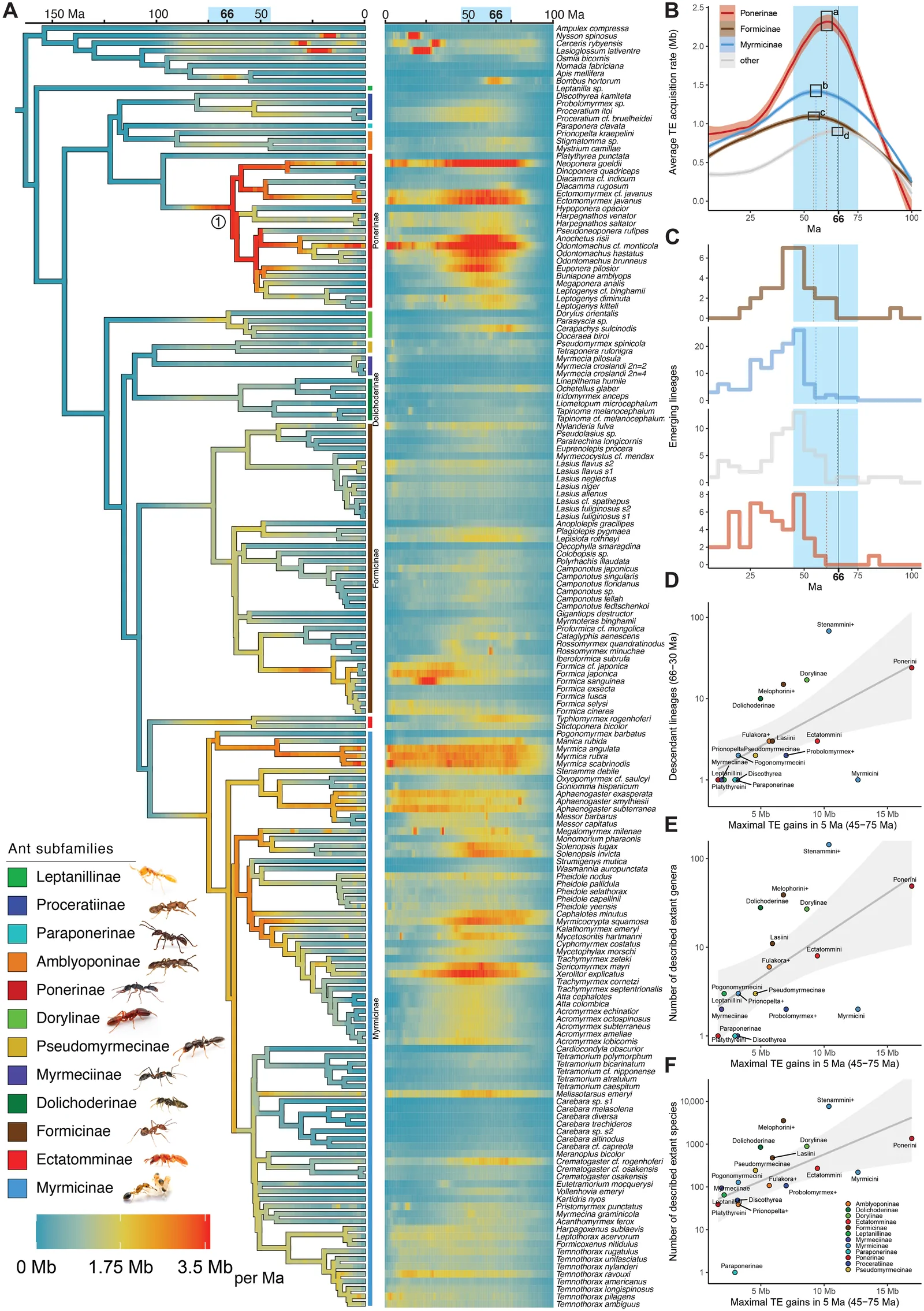
Evolutionary history of TE proliferation and lineage diversification. (**A**) Heatmap-colored phylogenetic tree (Fig. 1A) showing TE insertion histories over the past 100 Ma. Periods of intensified TE proliferation show up in red (up to 3.5 Mb/Ma). Branches were color-coded according to estimated TE gains per 1 Ma. The light blue rectangles along the *x* axes (top) highlight the crucial period of high TE acquisition 45 to 75 Ma. (**B**) The averaged TE proliferation history for the three largest and the pooled nine other ant subfamilies. Lighter colored shadings are 95% CI. TE proliferation was strongest 45 to 75 Ma for all subfamily categories (light blue column) with letters indicating significant differences (adjusted *P* < 0.05) between 5-Ma peaks of TE sequence gain (black rectangles) according to Bonferroni-corrected Dunn’s test. (**C**) Lower taxonomic lineages as they emerged over time (in bins of 5 Ma) in the Ponerinae, Myrmicinae, Formicinae, and other ant subfamilies 10 to 100 Ma, with most new lineages arising in the first half of the Paleogene (35 to 66 Ma). The K-Pg boundary 66 Ma is indicated by a blue solid line, while colored dashed lines indicate TE activity peaks (Fig. 2C). (**D** to **F**) For ant lineages that existed at the K-Pg boundary at 66 Ma, regression analyses showed that the maximal amount of TE sequences gained per 5-Ma interval between 45 and 75 Ma (*x* axis) explains ∼30% of the variation in the number of lineages subsequently descending from these branches between 35 and 66 Ma [D, *pgls*: TE_gains ∼ log_10_(descendant_lineages + 1), *P* < 0.008, adj. *R*^2^ = 0.333] (*45*), the number of extant genera [E, *pgls*: TE_gains ∼ log_10_(extant_genera), *P* < 0.008, adj. *R*^2^ = 0.326], and the number of known species [F, *pgls*: TE_gains ∼ log_10_(extant_genera), *P* < 0.015, adj. *R*^2^ = 0.275]. Regression lines (in gray) were derived from *pgls* models. Gray shadings show 95% CI. See the Supplementary Materials and fig. S7.

To study deep-time TE acquisition dynamics, we developed a novel approach to modeling the history of TE proliferation in a phylogenetic context using Kimura distance–based dating. Estimations of TE insertion age derived from such divergence-from-consensus approaches correlate well with phylogeny-informed estimates (*42*), so the Kimura model represents a robust proxy of past TE activity (see also fig. S5) (*43*). We used Kimura distances (*44*) between individual TE copies and their corresponding TE consensus sequence and mutation rates, estimated for each subfamily of ants, to bin TEs by insertion age. We then modeled past TE insertions at internal branches across the phylogeny by computing the 90% quantile of insertion ages inferred from the corresponding bins of extant species (Fig. 2A and fig. S6). We found that during the past 100 Ma, most TE insertions occurred 45 to 75 Ma, overlapping with the K-Pg boundary at ∼66 Ma, a result particularly evident for the largest subfamilies Ponerinae, Formicinae, and Myrmicinae (Fig. 2B and fig. S7). The most notable signature of high TE insertion activity occurred in the common ancestor of the tribe Ponerini (∼60 Ma; Fig. 2B, label ① in Fig. 2A).

We used data from a recent phylogenetic study covering 277 (81%) of the recognized extant ant genera to reconstruct how subfamilies of ants diversified over time (*45*). This lineages-through-time analysis revealed that most diversification happened in the first half of the Paleogene (∼35 to 66 Ma), i.e., after the inferred periods of maximal TE insertion activity (Fig. 2C). We then tested whether the inferred rates of TE acquisition spanning the transposition peaks 45 to 75 Ma could predict the extent to which different ant lineages diversified subsequently. Focusing on the phylogenetic branches present at the K-Pg boundary ∼66 Ma (fig. S8), we used *pgls* models to assess correlations between the maximum TE sequence gain rate within each of the six 5-Ma windows (i.e., using the amplitudes of the TE acquisition rates as in Fig. 2C) and (i) the number of descendant lineages originating in the early Paleogene (Fig. 2D), (ii) the number of extant genera (Fig. 2E), and (iii) the number of extant species (Fig. 2F) arising from these branches. Consistent with the hypothesis that TE expansions facilitated the evolutionary diversification of lineages, we found highly significant correlations in each of these cases.

### TE-associated gene family expansions and their social evolution implications

**Fig. 3.**
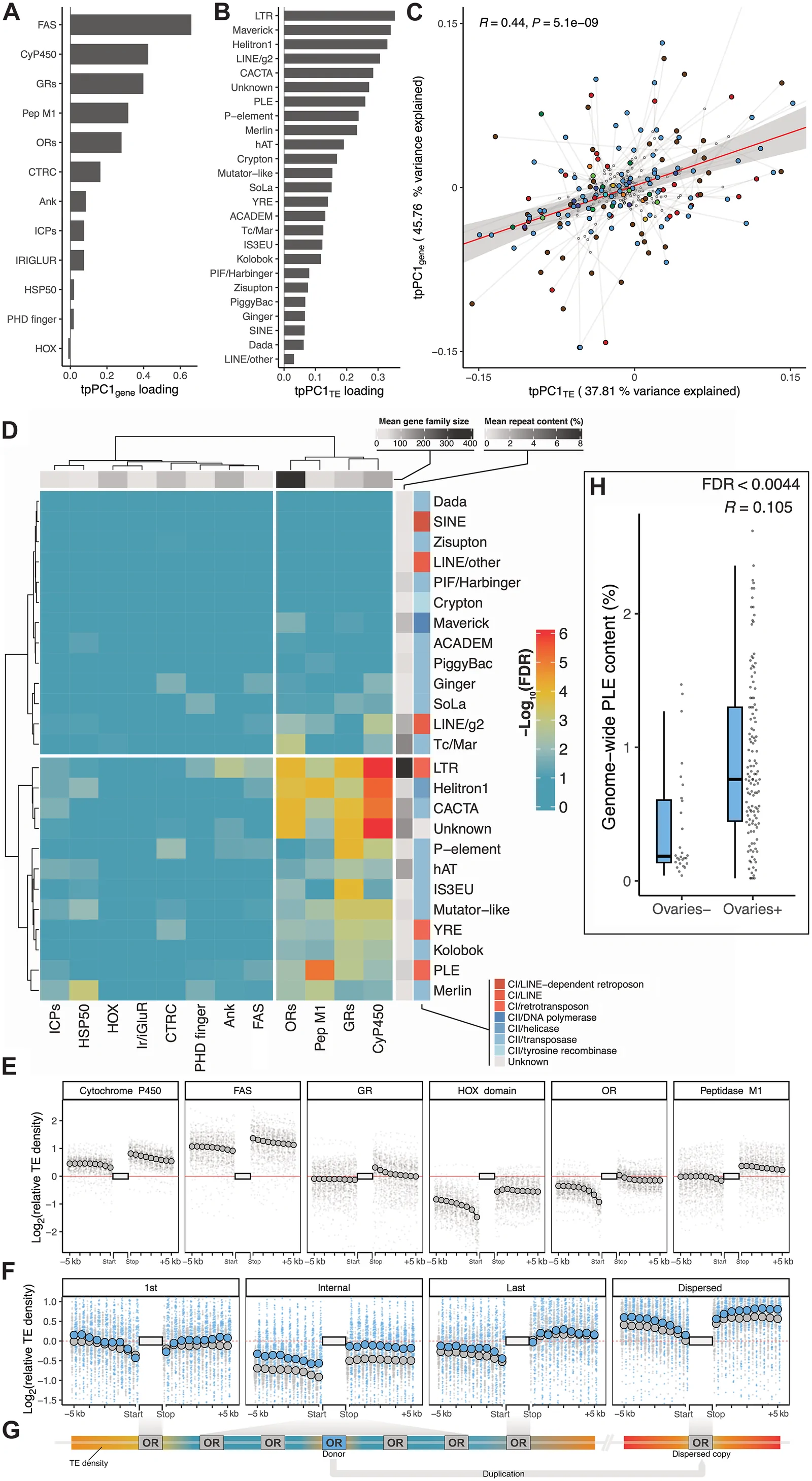
Genomic and phenotypic correlates of TE content. (**A** and **B**) First principal components loadings of copy number variation for the 12 largest gene families (A, tpPCA_gene_; fig. S9) and for variation in genome-wide abundance of different TE orders (B, tpPCA_TE_; fig. S10) based on transformed phylogenetic PCA. (**C**) Correlation of tpPC1_TE_ (*x* axis) and tpPC1_gene_ (*y* axis) shown as a red line (95% CI in gray). Colored dots show tpPC1_TE_ and tpPC1_gene_ coordinates for different taxa. Superimposed phylogenetic relationships are illustrated by gray lines (fig. S11). (**D**) Correlation between genome-wide abundances of TEs (rows) and gene family sizes (columns), visualized in a heatmap showing FDR values from *pgls*. Mean gene family sizes and mean genome-wide content for different TEs shown in gray. Polychromatic heatmap rows and columns formed four major groups after Ward’s clustering. The bottom-right cluster highlights significant correlations between multiple TE orders and ORs, PepM1s, GRs, and CyP450s. (**E**) Normalized TE densities for CyP450, FAS, GR, HOX domain, OR, and PepM1 genes. Gray circles represent average, normalized TE densities at 500-bp intervals for 4.5 kb up- and downstream of focal genes. (**F**) Increased TE density of dispersed ORs and their paralogs in ant genomes, plotted as normalized TE densities for ORs separated by their position within tandem arrays or outside of tandem arrays (dispersed copy). Point colors separate direct paralogs of dispersed ORs (“donors” in blue) from other ORs (gray). Background jitter plots in (E) and (F) show average normalized TE densities per genome. Rectangles represent focal genes. (**G**) Illustration of TE density variation in ORs, with dispersed copies originating via duplication of paralogous donors (in blue) and TE densities shown by shades of red (high) and blue (low). (**H**) Genomes of species with sterile workers (ovaries−) contain significantly fewer PLEs than species where workers have retained ovaries (ovaries+; fig. S17).

The proliferation of TEs is a mutagenic process that can affect the functional genomic repertoire of protein-coding genes (*46*). Therefore, we tested whether the evolution of copy-rich gene families in ants is associated with TE content. For this, we conducted two transformed phylogenetic principal component analyses (tpPCAs) (*47*) to identify covariances between copy number variation across different gene families (tpPCA_gene_) and the underlying genome-wide variation in TE content among ant species (tpPCA_TE_) while also accounting for phylogenetic relationships between the included taxa. In tpPCA_gene_ (fig. S9) we analyzed the 12 largest gene families (average copy number > 30; Fig. 3A), while in tpPCA_TE_ (fig. S10) we examined the genome-wide content of the most abundant TE orders (average content > 0.01%, across 6 class I and 18 class II orders; Fig. 3B).

PC1 of tpPCA_gene_ (explaining ∼46% of gene copy number variation) and PC1 of tpPCA_TE_ (explaining ∼38% of TE content variation) were highly significantly correlated (Pearson *r* = 0.44, *P* = 5.1 × 10^−9^; Fig. 3C and fig. S11), indicating that variation in TE content accounts for a substantial fraction of the cross-species variation in several key gene families. We next used phylogenetically corrected *pgls* models [correcting for false discovery rate (FDR)] to test whether copy number variation in each of the 12 gene families was correlated with the genome-wide abundance of different major TE orders. Four gene families showed particularly strong associations with important TE orders: ORs and gustatory receptors (GRs), which are crucial for chemical perception and communication (*41*), CyP450s involved in cuticular hydrocarbon biosynthesis and various other functions (*48*), and peptidase M1 membrane alanine aminopeptidases (PepM1s), which are implicated in diverse physiological functions but remain uncharacterized in ants (Fig. 3D and figs. S12 and S13). Eight other gene families, including homeodomain proteins (HOX) and ionotropic/ionotropic glutamate receptors (IR/iGluR), showed no such significant correlations with TEs.

To further explore differences in the association between TEs and gene family expansions over evolutionary time, we compared the abundance of TEs across 500-bp sliding windows 4.5 kb up- and downstream of copy-rich gene families (Fig. 3E and fig. S14). HOX genes had substantially reduced flanking TE content, consistent with TE insertions being likely to be removed from the vicinity of these highly conserved genes (Fig. 3E) under strong purifying selection (*49*, *50*). In contrast, we found elevated TE densities around CyP450s and downstream of PepM1 genes (Fig. 3E), indicating that transpositions are more common in the proximity of these genes. We also found high TE densities around FAS genes, although copy number variation was uncorrelated with genome-wide TE content (Fig. 3D), indicating distinct evolutionary dynamics in this gene family.

Against our expectations, neither ORs nor GRs had elevated TE densities in their flanking regions (Fig. 3E), but TE densities were enhanced up- and downstream of OR and GR tandem arrays and particularly around dispersed OR (Fig. 3F) and GR copies (fig. S15), i.e., genes isolated from other ORs/GRs and flanked by non-OR/GR genes. To test whether enrichment around dispersed copies reflects their origin via TE-mediated duplication (*51*), we analyzed TE densities for direct paralogs (i.e., the most likely templates of dispersed duplicates) (Fig. 3F and fig. S15). Genes most similar to dispersed OR/GR gene copies had flanking regions with elevated TE densities, consistent with an increased likelihood of these genes having originated as dispersed duplicates mediated by TEs (Fig. 3G) (*52*).

Leveraging social evolution and ecology data collected and partly analyzed by Vizueta *et al.* (*7*), we tested for potential associations between the prevalence of different orders of TEs (>1% of the average genome-wide content) and a number of phenotypic ant traits (fig. S16). We detected only one significant association after correcting for phylogenetic confounders and multiple testing (*pgls* models, FDR < 0.01), namely, that the genomes of ants with fully sterile workers [an evolutionarily derived trait that evolved convergently across a number of ant genera (*7*, *53*)] contained significantly fewer PLE retrotransposons (Fig. 3H and fig. S17). In *Drosophila virilis*, PLEs are known to cause dysgenesis (*54*), producing phenotypes that are remarkably reminiscent of sterile ant workers, as dysgenetic *D. virilis* females suffer from gonadal atrophy, germ cell loss, and inability to produce eggs. This coincidence offers the hypothesis that PLEs may originally have been co-opted to suppress reproduction by ant workers but that this function became obsolete in lineages where workers lost their ovaries, so PLEs were no longer maintained by selection either. Molecular studies comparing PLE expression in active and inactive worker ovaries will be needed to test this hypothesis.

### Prevalent patterns of genome compartmentalization in ants

**Fig. 4.**
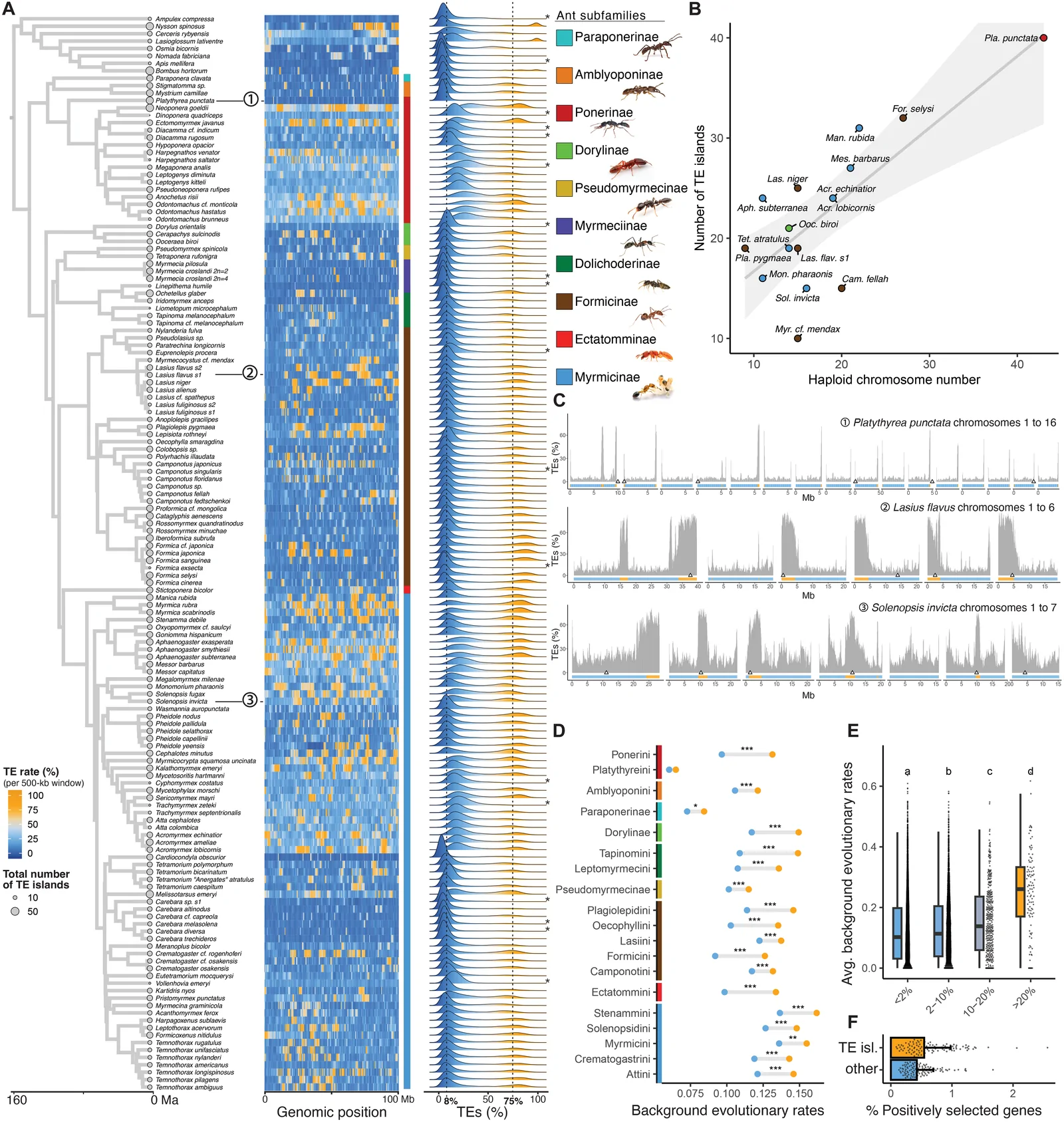
TE-driven compartmentalization of ant genomes. (**A**) Prevalence and extent of TE compartmentalization in ant genomes. Point sizes at terminal branches of the phylogeny (*7*) indicate TE islands per genome (in scaffolds >2.5 Mb). Labels ① to ③ highlight genomes detailed in (C). The heatmap shows TE content across 500-kb windows for the most contiguously assembled 100 Mb per genome, revealing a pattern of low-TE regions (<30%, dark blue) punctuated by TE-rich TE island clusters (>60%, orange). Note that TE-rich regions are more prone to assembly gaps and may thus be underrepresented in the most contiguous segments we visualize here. Ridge plots show the characteristic bimodal TE distribution (peaks below 30% and above 60%). Asterisks mark 18 genomes lacking statistical support for bimodality. Dashed lines indicate average low-TE (8%) and high-TE (75%) peaks. (**B**) Haploid chromosome number (*x* axis; table S3) predicts TE island count (*y* axis) in 16 chromosome-level assemblies (linear model fit 95% CI). (**C**) TE content along the largest chromosomes of *P. punctata* (①), *L. flavus* (②), and *S. invicta* (③), with predicted centromeres marked by small triangles. Orange highlights indicate annotated TE islands. (**D**) Genes in TE islands show elevated evolutionary rates compared to TE-poor regions. Dumbbell plots show average rates by ant tribe and subfamily. Asterisks indicate significant differences (Wilcoxon tests; */**/*** = FDR < 0.05/0.01/0.001). (**E**) Evolutionary rates increase with TE island occupancy. Jitter plots show median evolutionary rates per orthogroup against the proportion of orthologs in TE islands across 105 genomes. Letters denote significant pairwise differences (Wilcoxon test; FDR < 0.05). (**F**) TE island genes show higher rates of positive selection. Bar plots show mean percentages of positively selected genes in TE islands versus other regions across 105 genomes (whiskers = SD).

The generally high recombination rates of ant genomes (*55*) may have been an important driver in shaping the chromosomal distribution of TEs, leading to an enrichment of TEs in nonrecombining regions and depletion of TEs in recombining regions (*56*). Evaluating this across the 137 ant genomes for which contiguous assemblies (*N50* > 1 Mb) were available, we found bimodal distributions of TE content in 122 cases (modality analyses; see the Supplementary Material), with genomic windows either containing 5 to 30% (median = 8%) or above 60% TEs (median = 75%) (Fig. 4A, right). Within the most contiguous parts of the assemblies (scaffolds over 2.5 Mb), we identified between zero (*Dinoponera quadriceps*) and 44 (*Neoponera goeldii*) regional compartmentalizations into distinct TE islands (using changepoint modeling; see Materials and Methods), with an average of 20 such TE islands per species (Fig. 4A and fig. S18). We also found TE islands in some Apoidea outgroup species, suggesting that genome structuring into distinct TE compartments is not restricted to ants.

Among the 16 ant species for which chromosome-resolved assemblies were available (table S3), the number of TE islands was significantly correlated with chromosome numbers (*pgls* model, *P* < 0.0006, adj. *R*^2^ = 0.56; Fig. 4B). Consistent with TE islands often emerging in nonrecombining, (peri-)centromeric regions, we also found a significant overrepresentation of centromeres (predicted with CentroMiner; see the Supplementary Materials at https://doi.org/10.17894/ucph.bec65ee0-77c7-4a2a-9ed1-c7b75029040c) in TE islands (Fisher’s exact test, *P* = 1.5 × 10^−13^, odds ratio = 4.57).

Assessing distributional variation in TE content across the available ant genomes revealed that the extent and discreteness of TE accumulation can vary considerably (Fig. 4C and fig. S19), from discrete short regions in *Platythyrea punctata* to large chromosome-spanning accumulations in *Lasius flavus* to more diffuse distributions in *Solenopsis invicta*, with individual chromosomes showing variable numbers of TE islands or none at all. TE-dependent genome compartmentalization has previously been linked to the emergence of “two-speed” genomes in fungi (*57*, *58*), where TE-rich parts evolve significantly faster than TE-poor parts. To test whether a similar pattern occurs in ants, we compared background evolutionary rates (using the first ω rate class fitted to most of the coding sequence; see Materials and Methods) between single copy ortholog protein-coding genes located in the two different types of genome compartments. This showed that evolutionary rates in TE-rich regions exceeded those observed in TE-poor regions in all ant clades (Fig. 4D and fig. S20). Consistent with this finding, orthogroups where more than 20% of the orthologs were located in TE islands had the highest average evolutionary rates with an accelerating trend (Fig. 4E). Gene Ontology (GO) term enrichment analyses further revealed that genes involved in responses to environmental cues (e.g., sensory perception, synaptic transmission, and fatty acid biosynthesis) were overrepresented in TE islands (fig. S21).

Together, these findings are in agreement with a model of ant genome architecture shaped by accelerated evolution of genes in TE-rich regions, most likely owing to relaxed evolutionary constraint (*39*, *56*). To probe whether the increases in evolutionary rate could have systematically contributed to the emergence of adaptive variants, we compared the frequency of genes showing significant signatures of positive selection in TE-rich and TE-poor compartments, using adaptive branch-site random effects likelihood (aBSREL) models implemented in HyPhy. Positive selection was significantly more frequent in genes located in TE islands (Fig. 4F; Wilcoxon test, *V* = 3920, *P* = 0.0003), consistent with a relatively higher frequency of adaptive innovations in TE-rich genomic regions compared to other compartments of ant genomes.

## DISCUSSION

Our results offer in-depth insight in the impact of TEs on the evolutionary history of the ants and their role in shaping genome architecture, gene family evolution, and adaptive radiation at lower taxonomic levels. We show that the evolutionary diversity of extant ants is tightly correlated with the abundance and proliferation of TEs in their genomes (Figs. 1 and 2), consistent with peaks of transposon activity preceding periods of rapid adaptive radiation (*59*) during the ants’ phylogenetic history in the Paleogene (Fig. 2) (*7*, *45*, *60*), particularly for the species-rich Myrmicinae and Ponerinae subfamilies (*61*). We note that our reconstruction of transposon activity was restricted to the past 100 Ma due to methodological constraints while the ants originated before the Cretaceous (*7*, *45*), so we cannot exclude the possibility of yet unknown TE bursts having affected the earliest history of ant diversification.

Increased transposon activity during periods of adaptive radiation has been documented in other lineages such as primates (*62*) and bats (*23*), where TEs likewise contribute to structural genome evolution and genetic innovation (*63*, *64*), consistent with our findings that TE expansions occurred in the first half of the Paleogene and affected the adaptive diversification of ORs, GRs, and other key gene families. These conclusions match known patterns of genome rearrangement in ants, as chromosomal breakpoint rates are also positively correlated with extant species diversity across subfamilies (*7*). Similar to the adaptive radiations of mammals (*65*), birds (*66*), and other vertebrate lineages, also the rapid diversification of the largest ant subfamilies followed in the wake of the major K-Pg extinction event ∼66 Ma (*60*). Increased activity of TEs during this crucial period may thus have been instrumental in mediating novel genetic variation and reproductive isolation (*13*, *15*, *67*, *68*) while facilitating the adaptive niche diversification of ants known to have happened at that time (*10*). Thus, TEs likely provided the raw genetic material facilitating diversification, while the ecological opportunities generated by the extinction event determined how this variation could become consequential.

We discuss our inferences in more detail below after first acknowledging key limitations of our study. Comparative genomic reconstructions rely on limited taxon sampling (e.g., we only cover 12 of the 16 known subfamilies of ants) and simplified molecular models, rendering them subject to uncertainty from sequencing quality, stochastic evolutionary processes, and potential systematic or technical biases. For instance, assembly quality and fragmentation of consensus models directly affect inferences of TE copy number, divergence, and distribution (*69*), while evolutionary models like Kimura’s necessarily oversimplify biological processes and cannot capture heterogeneities across time and lineages (*70*). Furthermore, our restriction to extant lineages means we cannot exclude the possibility that extinct taxa exhibited different patterns of TE activity, gene family dynamics, and diversification histories that might have altered our conclusions.

All evolutionary genomic studies are inherently limited in their ability to establish causal relationships, as they necessarily address events that occurred millions of years ago. The K-Pg extinction event, for example, could have independently promoted both lineage diversification and TE proliferation without direct causal coupling between them. However, our analyses were explicitly structured to test directional predictions, with TE accumulation preceding lineage diversification in time (Fig. 2, D to F), making reverse causality implausible. It would also not make sense to invert predictions in Fig. 1 (C and D), as lineage diversity cannot reasonably predict TE content. Similarly, gene family sizes cannot reasonably predict genome-wide TE content (Fig. 3C). While we cannot quantify the exact proportion of variance in our response variables attributable to TE-dependent processes, our results provide strong evidence that it represents a substantial component.

The associations between TE densities and expansions in several key gene families (Fig. 3) suggest that TE-dependent processes contributed substantially to these diversifications. While previous studies implicated TEs as drivers of gene family expansions in select ant species, particularly ORs (*39*, *71*), our broad phylogenetic sampling now allows generalizations across the ant subfamilies. These findings support the hypothesis that this invertebrate adaptive radiation has been shaped by TE acquisitions to a degree comparable to the vertebrate lineages discussed above, consistent with the well established general importance of TEs in driving genome evolution across insects more broadly (*72*–*76*).

It is notable that TE-associated gene family diversifications appear to have primarily affected the prevalence of specific focal adaptations related to ORs, GRs, and CyP450 (*7*), while more conserved gene families such as HOX proteins show signatures of purifying selection that would have precluded association with TE accumulations. These correlations indicate that the likelihood of purifying and/or positive selection differs between gene families, with some (e.g., HOX and IR/iGluRs) experiencing stronger purifying selection than others (ORs, GRs, and CyP450), and that TE-dependent gene duplications primarily become fixed by natural selection in gene families contributing to short-term adaptive evolution in ants.

The compartmentalization of genomes into TE-rich and TE-poor regions might constitute a general genomic mechanism to spur accelerating change in certain genomic compartments along with the genes contained within them (Fig. 4), independent of evolutionary stasis in other sections. A previous study on the evolutionarily derived ant *Cardiocondyla obscurior* (*39*) showed, for the first time, that distinct TE islands are prevalent across the genome of this species, a result that our present study shows to be a general phenomenon across the ant family. Similar patterns have been extensively described in several pathogenic fungi (*57*, *58*), and they might be important in other eukaryote lineages as well (*77*). The possible implications of such genomic substructuring may be quite fundamental, because they suggest a molecular basis for adaptive increases in evolutionary rate for specific genes and gene families located in TE-rich parts of the genome.

The genomic prevalence of TEs is determined by transposon activity, selection, and recombination (*10*, *11*, *78*), with nonrecombining regions such as (peri-)centromeres (*79*) are commonly enriched with TEs (*80*). Ants and other social Hymenoptera have extraordinarily high recombination rates, potentially increasing the extent to which TEs cluster in nonrecombining parts of the genome (*55*). In *C. obscurior* (*39*), TEs are remarkably rare in regularly recombining parts of the genome, while (peri)centromeric regions have become nearly saturated with TEs (*56*). As it turns out, the enrichment of TE islands around centromeres in ants (Fig. 4C) is consistent with centromeric TE accumulations in other lineages (*81*). However, centromere function in ants remains poorly understood and thus deserves further investigation to resolve how TE acquisition, recombination rate, and centromere evolution interact to produce such distinct patterns. 

Although the impact of TEs in organismal and genome evolution is now well appreciated, in depth comparative studies are still rare, for the repetitiveness, diversity, and short evolutionary half-lives of TEs make it difficult to reconstruct their impact over long evolutionary time spans. We have addressed this challenge by leveraging a phylogenetically broad set of highly contiguous genomes and curated TE libraries to pursue general conclusions regarding the role of TEs in the evolutionary history of the ants. We reconstructed the proliferation of TEs during 100 Ma of evolutionary history mapping their impact on adaptive radiation of the major ant subfamilies after the K-Pg extinction event (Figs. 1 and 2). Our analyses revealed that TEs are associated with gene family evolution and possibly downstream changes in social evolution (Fig. 3), and we uncovered broad-scale TE-dependent structural dynamics affecting the genomes of most ant species (Fig. 4). Similar studies on other eukaryotic lineages are now increasingly becoming feasible as larger numbers of high-quality genomes become available. Given the relationships that we could quantify for the ants, such future studies can be expected to substantially expand our general understanding of the macroevolutionary impact of TEs and their role in adaptive radiations across the tree of life.

## MATERIALS AND METHODS

### De novo identification, curation, classification, and annotation of TEs in ant genomes

TEs were annotated in the genomes of 163 ants and eight hymenopteran outgroup species. This set of genomes was compiled by the Global Ant Genomics Alliance (GAGA) and included species from 12 of 16 extant ant subfamilies (*7*). GAGA predominantly sequenced genomes using a combination of PacBio long-read and whole-genome short-read sequencing. Sixteen of the genome assemblies were improved to chromosome level by HiC sequencing. Another 33 ant genomes were assembled using short-read data, producing less contiguous genome assemblies compared to those based on long-read PacBio data. Lower-quality genomes were excluded from parts of the analyses where contiguity was particularly important. In addition to the genome assemblies and protein-coding gene annotations, GAGA also gathered phenotypic and life history data for the different ant species used in this study (see below). Detailed methods regarding sampling, data collection, sequencing, genome assembly, genome annotation, and phylogenetic inference are described in the study of Vizueta *et al.* (*7*).

We ran RepeatModeler2 v2.0.1 (*82*) with the option “-LTRStruct” to generate de novo repeat models for the genomes of the 163 ant and eight hymenopteran outgroup species included in this study. The resulting de novo repeats were curated with MCHelper (*83*) (in fully automatic mode, -a F, github.com/GonzalezLab/MCHelper/), which extends consensus repeat models by a “Blast, Extract, and Extend” approach, filters out putative false positives, classifies repeats by homology to dfam elements, evaluates structural features of TE consensus models, and reduces redundancy of the model library by clustering with CDhit. Initial assessment of the MCHelper-curated repeat libraries revealed persisting contaminations with protein-coding genes, in particular emerging from high copy number gene families. To purge these contaminations, we aligned repeat models against pfam domains with the pfam_scan.pl script (github.com/gpertea/gsrc/blob/master/scripts/pfam_scan.pl) and removed models with significant similarity to protein domains (*e*-value < 1 × 10^−5^) not typically found in TEs (see file TEdomains.pfam.tsv in zivgitlab.uni-muenster.de/schradel/GAGA-TEs/ for a whitelist of TE-associated domains). In addition, we removed repeat models showing significant homology to protein-coding genes annotated in ants based on alignments produced with mmseqs easy-search (alignment length > 100 bp, *e*-value > 1 × 10^−10^). We acknowledge that this filtering may have inadvertently removed some genuine TE-derived sequences when the underlying gene annotation itself contained residual TE contaminations, but we preferred specific correctness over comprehensiveness in our TE annotations. Models retained in the decontaminated and curated repeat libraries were classified with RepeatClassifier (*82*) and TEclass (*84*).

Following the above approach, we generated 171 curated and decontaminated de novo repeat libraries that were subsequently clustered and merged using mmseqs easy-cluster with the following options: --cov-mode 1 -c 0.8 --min-seq-id 0.8 -s 100 --threads 40 --exact-kmer-matching 1. This ant- and Hymenoptera-specific repeat library was then combined with curated models included in dfam3.7 followed by another round of clustering with mmseqs (same settings as above). On average, this pipeline yielded 56 DNA and 98 RNA TE models per genome. Most RNA TE models were classified as LTR elements (95%). For DNA elements, Tc1/Mariner (32%), hAT (18%), and CACTA (14%) elements were most common (Fig. 1B and figs. S1 and S2). Additional information about the different processing steps is provided in the code repository. We used the joint repeat library to annotate TEs in each genome. Initial repeat annotation was done with RepeatMasker v4.1.4 (www.repeatmasker.org/) and further polished using RepeatCraft (github.com/niccw/repeatcraftp), integrating structural annotations from LTRfinder (github.com/xzhub/LTR_Finder).

### Transformed phylogenetic principal components analyses

To correlate genome-wide copy number variation in different gene families with TE content across all ant genomes, we performed tpPCAs using the R function gm.prcomp with “GLS = T, align.to.phy = F, transform = T” as implemented in the geomorph library (*47*). We used the time-calibrated species tree by Vizueta *et al.* (*7*) in gm.prcomp to account for the nonindependence of included taxa due to their varying phylogenetic relationships. These tpPCAs condition the rotation of data space available in the phylogeny to obtain the principal components that capture variation in focal response variables independent of phylogenetic proximity. In the first tpPCA (tpPCA_gene_), we analyzed copy number variation [retrieved from Vizueta *et al.* (*7*)] among the 12 largest gene families, all with more than 30 copies per genome on average: insect cuticle proteins, IR/iGluRs, odorant receptors (ORs), GRs, homeodomain proteins (HOX), ankyrin repeat proteins (Ank), peptidase S1/S6 chymotrypsins (CTRC), PepM1, PHD finger-domain proteins, heat-shock protein 40s, CyP450, and FAS. In the second tpPCA (tpPCA_TE_), we examined the genome-wide TE content (in percentage) of the 24 most abundant TE orders represented in ant genomes (average content > 0.01%, six class I and 18 class II orders), based on the RepeatMasker TE annotations and classifications generated as described above: LINE/other, Dada, SINE, Ginger, PiggyBac, Zisupton, PIF/Harbinger, Kolobok, IS3EU, Tc/Mar, ACADEM, YRE, SoLa, Mutator-like, Crypton, hAT, Merlin, P-element, PLE, CACTA, LINE/g2, Helitron1, Maverick, and LTR.

### General linear modeling of genomic and phenotypic correlates of TE content

We used phylogenetic generalized linear models (*pgls*, with lambda = “ML”) implemented in the R library *caper* (github.com/davidorme/caper) to fit the genome-wide content of different TE orders (in percentage; see above) to different genomic (gene family copy numbers; see above) and phenotypic ant traits. Correlations were considered significant after correcting for multiple testing using Benjamini and Hochberg corrections at FDR < 0.01. For correlating genome wide TE content with phenotypic traits, we selected 14 traits for which at least 100 data points were available across the 163 ant species. As the biology of some of the included ant species remains understudied resulting in several missing data points, comprehensive multivariate analyses were not possible for these data. To obtain sufficient statistical power, we therefore required that fewer than 75 species shared the same trait value for discrete variables. This reduced the phenotypic dataset to eight variables (fig. S16): “colony size” (small, medium, large, or supercolonies), “polygyny” (i.e., queen number per colony; facultatively or obligately polygynous versus monogynous), “worker ovaries” (present or absent in the worker caste), “foraging stratum” (hypogaeic, epigaeic, or arboreal), “mean annual ambient temperature at the sampling site”, “diet specialization” (“predator,” “omnivore,” “herbivore,” and “fungivore”), “trophobiosis” (keeping Sternorryncha, i.e., predominantly aphids and coccids, or not), and “trophallaxis” (presence or absence of worker-worker liquid food exchange). All model summaries are included in the extended data repository at https://doi.org/10.17894/ucph.bec65ee0-77c7-4a2a-9ed1-c7b75029040c.

### TE density analysis

To quantify the presence of TEs relative to genes and assess a biased localization of TEs in relation to certain gene families, we used TE Density v2.3.1 (*85*). TE density is defined as the proportion of base pairs occupied by TEs relative to a given window size in the genome. We calculated TE density values per gene across 4.5 kb up- and downstream windows with 500-bp intervals. For all genomes, TE density was run with default parameters. TEs overlapping with annotated coding sequences were excluded from the analysis to remove protein-coding genes that were potentially misannotated as TEs. Small scaffolds not containing any annotated genes or TEs were also filtered out. During the preprocessing stage of the TE density pipeline, redundant TE order and superfamily groupings were collapsed, consistent with the guidelines of the TE density software (e.g., Kolobok-E and Kolobok-H to Kolobok).

Average TE density values were calculated for 12 different gene families of interest (see above for a description of those gene families), for other genes (“General”), and for the complete gene set per genome. Accordingly, we obtained 14 values per window and TE type for each ant species. To account for differences in TE content between species, average TE density values were normalized by dividing observed TE densities for a given gene family by the TE densities obtained for all other genes in a genome. These normalized TE density ratios were log_2_-transformed and plotted for each gene family, covering 5 kb upstream and downstream regions of genes.

For additional analyses, genes of OR and GR families were clustered into tandem arrays with bed_cluster (with max_dist = 10,000) from the valr R package (*86*). We next grouped genes into four categories: “first” and “last” for genes at the first and last positions in a tandem array, respectively (relative to genomic coordinates), “internal” for genes inside tandem arrays, and “dispersed copies” for genes not clustered into tandem arrays but located at genomic loci distant from other members of their gene family. For first, last, and internal gene sets, the upstream and downstream TE density was calculated relative to the tandem array and not relative to the strandedness of the respective genes.

We identified the putative closest paralogs of OR and GR dispersed copies with homology searches with mmseqs (v.15-6f452, github.com/soedinglab/mmseqs2) using the protein sequences of dispersed copy genes as queries. The highest scoring nonself hit (according to an *e*-value and a bitscore) for each dispersed copy was considered the closest paralog. The complete TE density pipeline applied to the ant genomes investigated in this study can be found at zivgitlab.uni-muenster.de/j_rink02/GAGA_TEs_Analysis with all instructions to run the code in a comparative, automatized framework.

### Kimura distance–based analysis of ancestral TE insertions

We used RepeatMasker’s calcDivergenceFromAlign.pl and parseRM.pl (-l 200,.5 --parse --age 1,10, from github.com/4ureliek/Parsing-RepeatMasker-Outputs) to compute Kimura distance–based estimates of insertion history for each TE family. The calcDivergenceFromAlign.pl script bins annotated TEs according to their Kimura distance to the respective consensus. To estimate the time since the split from the consensus based on the calculated Kimura distance with parseRM.pl, we estimated average neutral mutation rates per subfamily and outgroup by extracting fourfold degenerate (4d) sites from high-quality codon alignments (after filtering with HmmClean) of 8876 single-copy orthologs (see the “Evolutionary rate analysis” section below). These extracted 4d sites were concatenated into a final matrix of 824,860 4d sites across 163 ant species. To estimate branch lengths for the 4d site–based phylogeny, we then used the fixed tree topology from Vizueta *et al.* (*7*) and inferred branch lengths using IQ-TREE 2 (v2.2.0) with the following command: “iqtree2 -s 4d.phy -te GAGA_tree.nhx.” Subsequently, we estimated neutral mutation rates per year using the software r8s (v1.8.1), again using the tree topology from Vizueta *et al.* (*7*) with inferred 4d branch lengths. We fixed the root age as being 157 Ma and applied the 37 fossil calibrations used in the original phylogeny dating analysis (*7*).

The r8s estimation was performed with the following command: “r8s -b -f r8s.constrain.ct,” after which average neutral mutation rates could be calculated by averaging across the estimates for all branches for each subfamily and the outgroup species, respectively. This yielded the following very similar mean neutral mutation rate estimates in substitutions per 4d site per million years: Myrmicinae: 0.0033, Formicinae: 0.0032, Ponerinae: 0.0037, Ectatomminae: 0.0033, Dolichoderinae: 0.0033, Pseudomyrmecinae: 0.0034, Dorylinae: 0.0035, Proceratiinae: 0.0037, Amblyoponinae: 0.0036, Leptanillinae: 0.0035, Myrmeciinae: 0.0034, Paraponerinae: 0.0036, outgroup: 0.0036.

We multiplied neutral mutation rate estimates by two to approximate TE-specific mutation rates. This scaling is consistent with theoretical and empirical studies (*87*–*90*), primarily derived from mutation accumulation experiments showing approximately doubled single-nucleotide mutation rates in repetitive contexts in *Arabidopsis* (*91*) and *Caenorhabditis elegans* (*92*) compared to other genomic regions. The output of parseRM.pl for each genome was combined in a single analysis in R (v4.2.3), which aggregated all TE data, separately for each class, subclass, and family of TEs, across all genomes.

To assess the effect of consensus TE model fragmentation on insertion time estimates, we compared Kimura distances between complete and incomplete TE consensus models for all annotated copies across all genomes (fig. S22). The differences in Kimura distances between “complete” and “incomplete” models were generally small, suggesting that consensus model fragmentation did not have a strong influence on our estimated insertion times. The largest difference between putatively complete and fragmented models was observed for the LINE elements that make up ∼5% of the curated repeat library. Here, the average Kimura distance for complete models was 33.2% against 28.3% for incomplete models.

To test whether genome assembly contiguity could have systematically affected estimates of TE copy Kimura divergence in the consensus model, we correlated contig N50 (as a measure of assembly contiguity) with average divergence estimates across the annotated TEs from different orders in our dataset (fig. S23). This analysis did not produce significant correlations for any TE order, suggesting that assembly quality had not systematically biased the inferred insertion time estimates of TE copies. We further compared Kimura landscapes for class 1 and class 2 TEs across different genomes in the genus *Acromyrmex* (with assembly quality ranging from contig N50 < 0.01 Mb to >8 Mb), which did not indicate systematic effects of assembly contiguity on divergence estimates either (fig. S24).

Kimura distance–based repeat landscapes across all 171 species were visualized along the species phylogeny as heatmaps, where rows show different species, columns show different time points (in million years in the past), and colors indicate the sum of genomic sequences annotated as a TE of a particular insertion age per species in bins of 1 Ma. This visualization allowed for comparing insertion histories across time and phylogenetic context.

We further conducted ancestral state reconstruction-like inferences of Kimura-based TE insertions for the internal nodes of the phylogeny by dividing each internal branch into bins of 1 Ma. We then retrieved the corresponding data from the bins of descendant species and calculated the extent of TE insertions relative to these ancestral lineages at each time point. To account for deletions of TEs, we assumed the ancestral state at internal branches to be defined by the 90% quantile of values retrieved from the descendant species (see fig. S6 for reconstructions using 50, 75, and 100%).

To quantitatively assess periods of accelerated diversification in different ant subfamilies, we used a recent time-calibrated ant phylogeny comprising 277 (81%) of the known ant genera from Borowiec *et al.* (*45*). On the basis of lineages-through-time analyses performed with the R function ltt.plot() from the package ape, we calculated the number of lineages gained in 5-Ma bins for the different higher-level branches of the ant phylogeny. Our reconstructions revealed that most of the TE insertions occurred 45 to 75 Ma, overlapping with the Cretaceous-Paleogene boundary at 66 Ma (Fig. 2A and fig. S6). To correlate these ancient TE proliferation peaks with the extent of later lineage diversification, we first retrieved branches from the K-Pg boundary 66 Ma from the phylogeny by Vizueta *et al.* (*7*) and then calculated the maximum amount of TE sequence gained in any 5-Ma window during the time period between 45 and 75 Ma in these derived or corresponding ancestral branches (fig. S8). Next, we identified the corresponding branches in the phylogeny by Borowiec *et al.* (*45*) and calculated the number of descendant lineages emerging from these branches (fig. S8). Last, we used phylogenetic generalized linear models (pgls, with lambda = ML) as described above from the R library caper to fit phylogenetically corrected linear models between inferred TE sequence gains and the number of lineages descending between 35 and 66 Ma ago across the branches [transformed to log_10_(descendant lineages + 1)]. Similarly, we tested for significant correlations between the inferred TE sequence gains and the (log_10_-transformed) number of extant described genera or species (retrieved from antwiki.org, accessed 12th of July 2025).

### Analysis of genomic compartmentalization of TE distributions

We investigated the genome-wide distribution of TEs by sliding window analyses, using only the highly contiguous assemblies of 137 (of 163) genomes with a scaffold N50 > 1 Mb. We calculated TE and exon content in nonoverlapping 50-kb windows using bedtools and bedmap (*93*). The distribution of TEs and protein-coding genes in the 10 largest scaffolds of each genome was then visualized and inspected, revealing notable patterns of nonrandom TE distributions in many of the genomes, where continuous segments of the scaffolds were either highly enriched with TEs or nearly depleted of TEs.

To comprehensively assess the extent of genome compartmentalization in the ant and outgroup genomes, we focused on the distribution of TEs in full genome assemblies, which allowed us to calculate mean TE content across 200-kb sliding windows for all scaffolds larger than 250 kb. To identify larger continuous regions enriched with TEs (“TE islands”), we used modeling implemented in the R package changepoint (*94*), particularly the “meancpt” model of the function envcpt (with options minseglen = 2, penalty = “Manual,” pen.value = 40), which fits one or multiple changepoints in the means and variances of continuous data. We then annotated TE islands as regions characterized in the changepoint models by an increase in TE content of at least 20% compared to either the TE content in neighboring regions or the scaffold-wide or genome-wide average TE content. The genome-wide distribution of TEs, exons, model-derived changepoints, and annotated TE islands was then visualized in summary plots for each species (see https://doi.org/10.17894/ucph.bec65ee0-77c7-4a2a-9ed1-c7b75029040c).

Manual reviewing of these plots confirmed the overall robustness of our TE island identifications as distinct genomic regions substantially enriched in TEs. However, we emphasize that the considerable biological diversity in TE content and distribution across the genomes limits the accuracy of this automatic “one-size-fits-all” approach. For analyses at the level of individual genomes, manual curation of TE island boundaries might thus be necessary. To identify centromere positions in the available 16 chromosome-level genome assemblies, we ran CentroMiner (github.com/aaranyue/quarTeT) with default parameters and created bed files with the best candidate per scaffold for each species, allowing us to investigate the extent to which centromeres were predicted in TE islands.

### GO enrichment analysis

We performed GO term enrichment analyses for all genomes (again excluding small scaffolds of less than 1 Mb), comparing the set of genes located in TE islands to the remainder of the genome. We first removed genes showing significant homology to known TEs with RepeatProteinMask (with -noLowSimple -engine ncbi), before defining the test gene sets based on overlap with TE islands. We then used topGO (*95*) for assessing significant enrichment of GO terms in TE island genes compared to the background gene set (*P* < 0.05, according to the topGO’s “parentChild” test). To assess convergent functional enrichment across species, we then compiled lists of GO terms showing significant enrichment at *P* < 0.001. This set of GO terms was analyzed further and visualized with simplifyenrichment in R (*96*) using kmeans clustering and annotation data from the fruit fly (org.Dm.eg.db_3.18.0).

### Evolutionary rate analysis

We used OrthoFinder v2.5.4 to infer orthology across the protein-coding genes of the 163 ant genomes. The coding sequences for each orthogroup were aligned using PRANK v170472 (-codon -F parameters), and the multiple sequence alignments were evaluated with Zorro. Orthogroups with average quality below 4 across more than 75% of the aligned positions were filtered out, and GARD was used in HyPhy v2.5.38 to identify recombination breakpoints and to split the alignments into separate partitions. HmmCleaner was then applied to further evaluate and curate the alignments, masking putative errors as gaps and removing codons with gaps in more than 50% of the species and sequences with fewer than 15 retained codons. A maximum likelihood tree was then constructed for each MSA partition using IQ-TREE. Using branch-site models, we tested whether genes in TE-rich compartments showed differences in evolutionary rates and an increased prevalence of positive selection signatures while limiting the analysis to genomes with a scaffold N50 > 1 Mb. Specifically, we used the aBSREL (*97*) model in the HyPhy package to infer evolutionary rates and detect signatures of positive selection across all branches in each partitioned orthogroup gene tree, based on high-confidence codon alignments.

aBSREL infers the optimal number of ω rate classes for each branch and assigns each site in the alignment to one of these classes, allowing different branches to feature evolutionary patterns of varying complexity. To compare background evolutionary rates of genes in TE-rich and TE-poor compartments, we extracted and compared (using FDR-corrected Wilcoxon tests) the ω1 rate class obtained from the model fits. In aBSREL, the ω1 rate class is commonly fitted to most of the coding sequence and was therefore considered as specifying a gene’s background evolutionary rate also in our analyses. To avoid spurious results, we excluded comparisons between species that diverged less than 5 Ma, excluding alignments where divergences between orthologous coding sequences were invariably low (reducing the set of included genomes to 105). We further restricted our analyses to genes with ω1 < 1 and to genes where ω1 could be fitted to at least 90% of the coding sequence (i.e., genes most likely to be subject to purifying selection for the vast majority of the gene). To analyze whether average evolutionary rates of single- and multicopy orthogroups were correlated with their prevalences in TE-rich and TE-poor compartments, we compared background evolutionary rates for all branches in orthogroup gene trees, separating orthogroups into four different bins, depending on the proportion of orthologs located in TE-rich compartments (<2%, 2 to 10%, 10 to 20%, and >20%). Also here, we only included branches where ω1 < 1 and where ω1 covered at least 90% of the aligned sequences (i.e., 90% of the sites having ω < 1). To assess the extent to which TE-rich and TE-poor compartments differed in their likelihood of adaptive molecular evolution, we calculated the proportion of protein-coding genes showing significant signatures of positive selection according to aBSREL’s likelihood ratio tests (FDR-corrected *P* value < 0.05) in both types of regions of the genomes.
