# Supplementary material for: Transposable elements as evolutionary catalysts of ant macrodiversity
Source: Sci Adv. 2026 Sep 23;12(39):eaee0306. doi: 10.1126/sciadv.aee0306 (PMC13600161; doi:10.1126/sciadv.aee0306)
Supplement: Supplementary file 1 [file sciadv.aee0306_sm.pdf]

Science Advances
AAAS

Supplementary Materials for

Transposable elements as evolutionary catalysts of ant macrodiversity

Lukas Schrader *et al.*

Corresponding author: Lukas Schrader, lukas.schrader@uni-muenster.de

Sci. Adv. **12**, eaee0306 (2026)
DOI: 10.1126/sciadv.aee0306

The PDF file includes:

Supplementary Text
Figs. S1 to S24
Legend for table S1
Tables S2 and S3

Other Supplementary Material for this manuscript includes the following:

Table S1

S1. Supplementary Materials

S1.1 Correlation of average TE content and species- or genus-richness

We analyzed the correlation of average TE content and species- or genus-richness across the different ant subfamilies using the `pgls` function in `R`.

pgls model summary for log10(genus count) ∼ TEcontent:

```
Call:
pgls(formula = log10(df$generacount) ~ df$mean.total, data = cd,
    lambda = "ML")

Residuals:
      Min        1Q    Median        3Q       Max
-0.060660 -0.039748  0.002873  0.036028  0.056713

Branch length transformations:

kappa  [Fix]  : 1.000
lambda [ ML]  : 0.000
   lower bound : 0.000, p = 1
   upper bound : 1.000, p = 0.20997
   95.0% CI    : (NA, NA)
delta  [Fix]  : 1.000

Coefficients:
               Estimate Std. Error t value Pr(>|t|)
(Intercept)   -0.227615   0.572198 -0.3978  0.69914
df$mean.total  0.055746   0.023983  2.3244  0.04245 *
---
Signif. codes:  0 '***' 0.001 '**' 0.01 '*' 0.05 '.' 0.1 ' ' 1

Residual standard error: 0.04448 on 10 degrees of freedom
Multiple R-squared: 0.3508,  Adjusted R-squared: 0.2858
F-statistic: 5.403 on 1 and 10 DF,  p-value: 0.04245
```

pgls model summary for log10(species count) ∼ TEcontent:

```
Call:
pgls(formula = log10(df$speciescount) ~ df$mean.total, data = cd,
    lambda = "ML")

Residuals:
    Min        1Q    Median        3Q       Max
-0.139158 -0.029680  0.003079  0.059079  0.071861

Branch length transformations:

kappa  [Fix]  : 1.000
lambda [ ML]  : 0.000
lower bound : 0.000, p = 1
upper bound : 1.000, p = 0.13405
95.0% CI   : (NA, NA)
delta  [Fix]  : 1.000

Coefficients:
            Estimate Std. Error t value Pr(>|t|)
(Intercept)   0.297002   0.855280  0.3473  0.73560
df$mean.total 0.095772   0.035849  2.6716  0.02343 *
---
Signif. codes:  0 '***' 0.001 '**' 0.01 '*' 0.05 '.' 0.1 ' ' 1

Residual standard error: 0.06649 on 10 degrees of freedom
Multiple R-squared: 0.4165,Adjusted R-squared: 0.3581
F-statistic: 7.137 on 1 and 10 DF,  p-value: 0.02343
```

S1.2 Peaks in TE sequence gains in 5 My between 45-75 Mya in different groups of ants

Dunn's test comparing TE sequence gains between subfamilies for peak TE gains in 5 My between 45-75 Mya.

```
Kruskal-Wallis rank sum test

data: x and group
Kruskal-Wallis chi-squared = 174.9833, df = 3, p-value = 0
```

```
                          Comparison of x by group
                                (Bonferroni)
Col Mean-|
Row Mean |   Formicin   Myrmicin      other
---------+---------------------------------
Myrmicin |  -5.134568
         |    0.0000*
         |
   other |   3.656614   8.530662
         |    0.0008*    0.0000*
         |
Ponerina |  -9.794227  -6.669085  -12.19095
         |    0.0000*    0.0000*    0.0000*

alpha = 0.05
Reject Ho if p <= alpha/2
```

S1.3 PGLS models correlating lineage diversification with TE sequences gained between 45-75 Mya

We assessed the linear correlation between the maximum amount of TE sequence gained within 5 My between 45-75 Mya in those lineages of ants that were inferred to exist 66 Mya and the number of descendant lineages (35-66 Mya, transformed to log10(descendant lineages + 1)), extant genera (log10-transformed), and extant species using the `pgls` function in `R`.

pgls model summary for descendant lineages (35-66 Mya) ∼ maximal TE gains in 5 My (45-75 Mya):

```
Call:
pgls(formula = log10(final.df$lineages + 1) ~ (final.df$kimura),
    data = cd, lambda = "ML")

Residuals:
    Min        1Q    Median        3Q       Max
-0.084450 -0.018142 -0.004459  0.002716  0.077244

Branch length transformations:
```

```
kappa  [Fix]  : 1.000
lambda [ ML]  : 0.000
lower bound : 0.000, p = 1
upper bound : 1.000, p = 0.010708
95.0% CI   : (NA, 0.662)
delta  [Fix]  : 1.000

Coefficients:
                Estimate Std. Error t value Pr(>|t|)
(Intercept)     -0.001992   0.199852 -0.0100 0.992171
final.df$kimura  0.083602   0.027137  3.0808 0.007163 **
---
Signif. codes:  0 '***' 0.001 '**' 0.01 '*' 0.05 '.' 0.1 ' ' 1

Residual standard error: 0.03692 on 16 degrees of freedom
Multiple R-squared: 0.3723,Adjusted R-squared: 0.3331
F-statistic: 9.491 on 1 and 16 DF,  p-value: 0.007163
```

pgls model summary for extant genera ∼ maximal TE gains in 5 My (45-75 Mya):

```
Call:
pgls(formula = log10(final.df$lit_genera) ~ (final.df$kimura),
    data = cd, lambda = "ML")

Residuals:
    Min        1Q    Median        3Q       Max
-0.086913 -0.023676 -0.002862  0.019673  0.078921

Branch length transformations:

kappa  [Fix]  : 1.000
lambda [ ML]  : 0.000
lower bound : 0.000, p = 1
upper bound : 1.000, p = 0.033094
95.0% CI   : (NA, 0.909)
delta  [Fix]  : 1.000

Coefficients:
                Estimate Std. Error t value Pr(>|t|)
```

```
(Intercept)     0.180789   0.231259  0.7818 0.445771
final.df$kimura 0.095368   0.031401  3.0371 0.007848 **
---
Signif. codes:  0 ‘***’ 0.001 ‘**’ 0.01 ‘*’ 0.05 ‘.’ 0.1 ‘ ’ 1

Residual standard error: 0.04273 on 16 degrees of freedom
Multiple R-squared: 0.3657,Adjusted R-squared: 0.326
F-statistic: 9.224 on 1 and 16 DF,  p-value: 0.007848
```

pgls model summary for extant species $\sim$ maximal TE gains in 5 My (45-75 Mya):

```
Call:
pgls(formula = log10(final.df$lit_species) ~ (final.df$kimura),
    data = cd, lambda = "ML")

Residuals:
      Min        1Q    Median        3Q       Max
-0.152004 -0.019890 -0.002275  0.026066  0.095662

Branch length transformations:

kappa  [Fix]  : 1.000
lambda [ ML]  : 0.388
   lower bound : 0.000, p = 0.45992
   upper bound : 1.000, p = 0.26421
   95.0% CI   : (NA, NA)
delta  [Fix]  : 1.000

Coefficients:
                Estimate Std. Error t value  Pr(>|t|)
(Intercept)     1.551724   0.341117   4.549 0.0003286 ***
final.df$kimura 0.113676   0.041624   2.731 0.0147999 *
---
Signif. codes:  0 ‘***’ 0.001 ‘**’ 0.01 ‘*’ 0.05 ‘.’ 0.1 ‘ ’ 1

Residual standard error: 0.05819 on 16 degrees of freedom
Multiple R-squared: 0.3179,Adjusted R-squared: 0.2753
F-statistic: 7.458 on 1 and 16 DF,  p-value: 0.0148
```

S1.4 Mode analysis of TE content distribution in ant genomes

We used the `modetest` package in `R` to assess the modality of the TE content distribution in 50 kb sliding windows across ant genomes. Specifically, we used `modetest(data, method="SI", mod0=1)` to test whether the true number of modes is greater than 1 and `modetest(data, method="SI", mod0=2)` to test whether the true number of modes is greater than 2. For 122 of the 137 tested ant genomes, the statistical analysis showed that the true number of modes is greater than 1 ($p < 0.05$), without support for a true number of modes greater than 2, indicating bimodality of the TE content distribution.

In the following 14 genomes, unimodality could not be rejected ($p > 0.05$): *Megaponera analis*, *Diacamma cf. indicum*, *Oecophylla smaragdina*, *Diacamma rugosum*, *Carebara sp. s1*, *Dorylus orientalis*, *Cyphomyrmex costatus*, *Dinoponera quadriceps*, *Formica exsecta*, *Linepithema humile*, *Odontomachus brunneus*, *Vollenhovia emeryi*, *Wasmannia auropunctata*, and *Trachymyrmex zeteki*.

In one species (*Myrmecia croslandi* 2n=4), the mode suggested a multimodal distribution with 3 modes (`modetest()` with `mod0=1`: $p = 0.002$; `modetest()` with `mod0=2`: $p = 0.028$; `modetest()` with `mod0=3`: $p > 0.05$).

The median positions of the low-TE and the high-TE peaks at 8% and 75%, respectively, were calculated using the `locmodes()` function in `R`, based on those genomes where bimodality was statistically supported.

S1.5 PGLS model correlating chromosome number and TE island count in ants

pgls model summary for chromosome_number ∼ TEisland_count:

```
Call:
pgls(formula = karyotype.comp$data$islandcount ~ karyotype.comp$data$x3,
    data = karyotype.comp, lambda = "ML")

Residuals:
     Min       1Q   Median       3Q      Max
-0.85868 -0.10986  0.07149  0.25412  0.54973

Branch length transformations:

kappa  [Fix]  : 1.000
lambda [ ML]  : 0.000
   lower bound : 0.000, p = 1
   upper bound : 1.000, p = 0.00030706
   95.0% CI   : (NA, 0.563)
delta  [Fix]  : 1.000
```

```
Coefficients:
                       Estimate Std. Error t value  Pr(>|t|)
(Intercept)             9.54487    3.16768  3.0132 0.0093047 **
karyotype.comp$data$x3  0.71575    0.16000  4.4733 0.0005254 ***
---
Signif. codes:  0 ‘***’ 0.001 ‘**’ 0.01 ‘*’ 0.05 ‘.’ 0.1 ‘ ’ 1

Residual standard error: 0.418 on 14 degrees of freedom
Multiple R-squared: 0.5884,Adjusted R-squared: 0.559
F-statistic: 20.01 on 1 and 14 DF,  p-value: 0.0005254
```

S1.6 Pairwise Wilcoxon rank sum test for differences in evolutionary rates between orthogroups

We compared the evolutionary rates within orthogroups relative to the proportion of orthologs found in TE islands using a pairwise Wilcoxon rank sum test.

```
Pairwise comparisons using Wilcoxon rank sum test with continuity correction

data:  omega.byHOG.df$bin.median.omega and omega.byHOG.df$proportion.bin

       <2%    2-10%  10-20%
2-10%  <2e-16 -      -
10-20% <2e-16 <2e-16 -
>20%   <2e-16 <2e-16 <2e-16

P value adjustment method: fdr
```

S1.7 Wilcoxon rank sum test for prevalence of positive selection in TE islands vs non-TE islands

We compared the prevalence of positive selection in TE islands vs non-TE island genes using a Wilcoxon rank sum test. For this, we calculated the percentage of genes showing positive selection in both genomic regions. We next calculated the log2-transformed ratio of these percentages for each genome and performed a Wilcoxon rank sum test to test for a significant deviation from 0 (i.e. no overall difference in the prevalence of positive selection between TE and non-TE islands across all genomes).

```
Wilcoxon signed rank test with continuity correction
data:  log2(absrel_df_leafs_sign_2$ratio)
```

```
V = 3449, p-value = 0.03325
alternative hypothesis: true location is not equal to 0
```

S2. Supplementary Figures

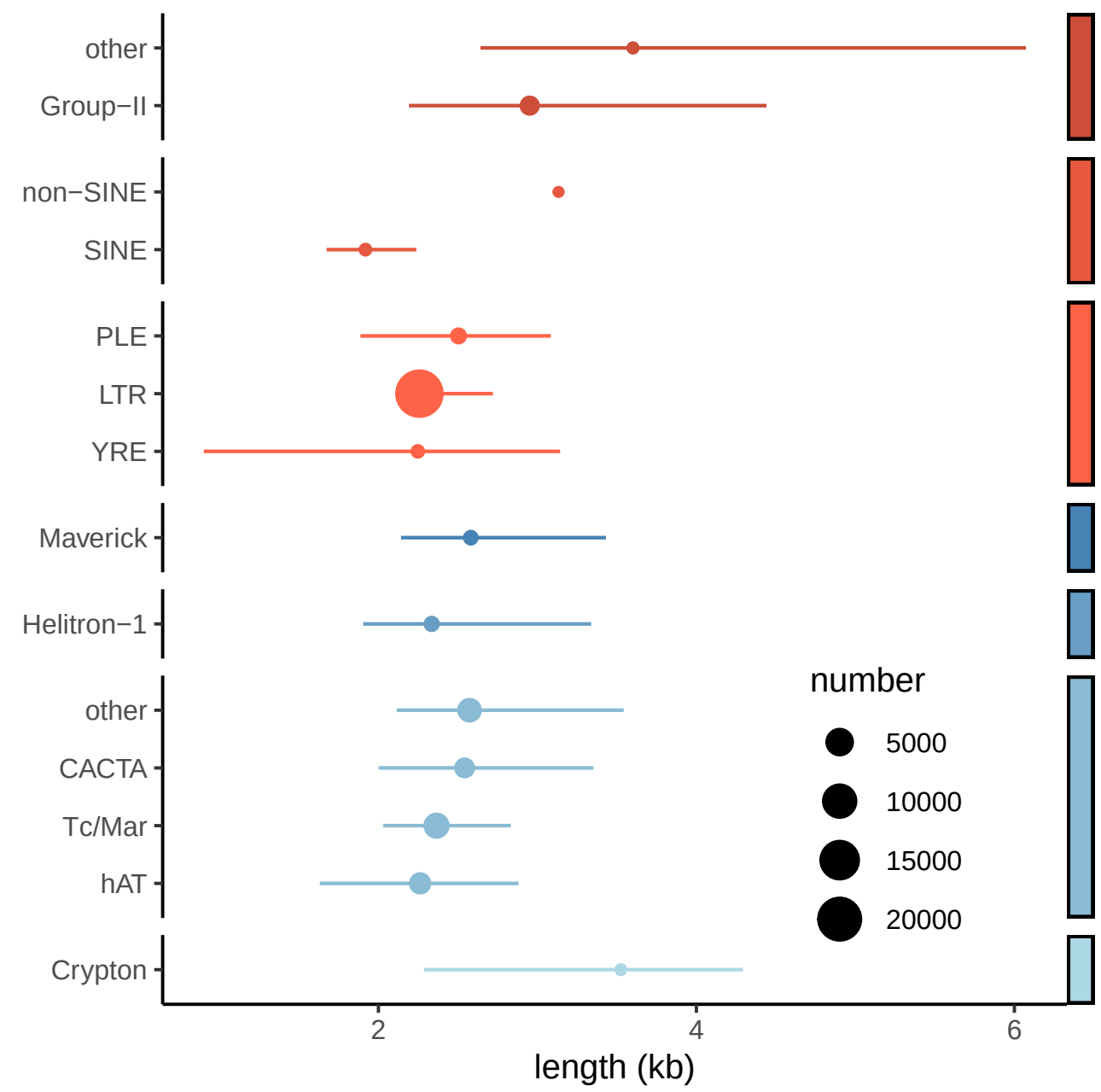

Figure S1: Composition of the joint de novo repeat library. For each TE order, the x-axis shows the length distribution in the joint consensus repeat library used to annotate the 171 genomes. The dots represent median lengths and whiskers show 25% to 75% quantiles of the length distribution. Dot sizes reflect abundances relative to the total number of each different type of TE in the final library. Red and blue shades separate TE subclasses according to the color coding in Figure 1. From top to bottom: In red: Class 1 LINE, Class 1 LINE-dependent retroposon, Class 1 retroposon. In blue: Class 2 DNA transposon, Class 2 Helicase transposons, Class 2 Transposase transposons, Class 2 Tyrosine Recombinase transposons. Only 18% of the classified consensus repeat models in the final library were identified as "complete" by `MCHelper` (8064 out of 43901), reflecting the limitations of consensus-based approaches for the reconstruction of full-length TEs in these ant genomes. See also Supplementary Figure S22.

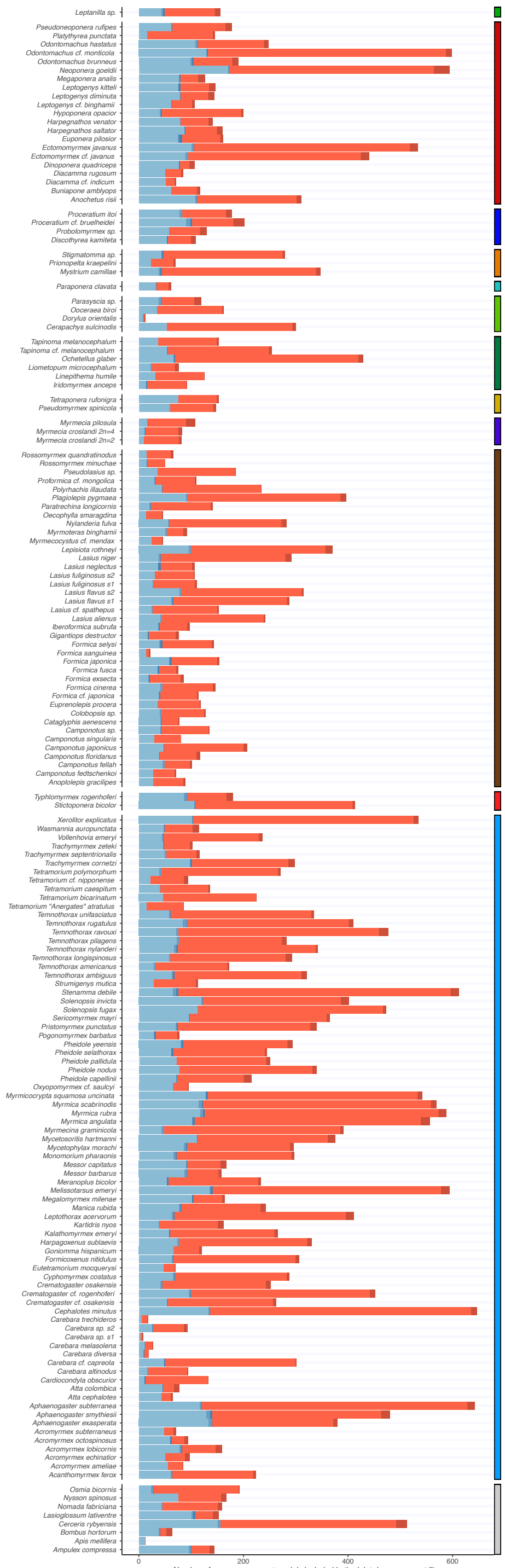

Figure S2: Number of consensus repeat models retained in the final joint library, separated by species. Colors for ant subfamilies (right side bar) and colors for TE subclasses are the same as in Figure S1.

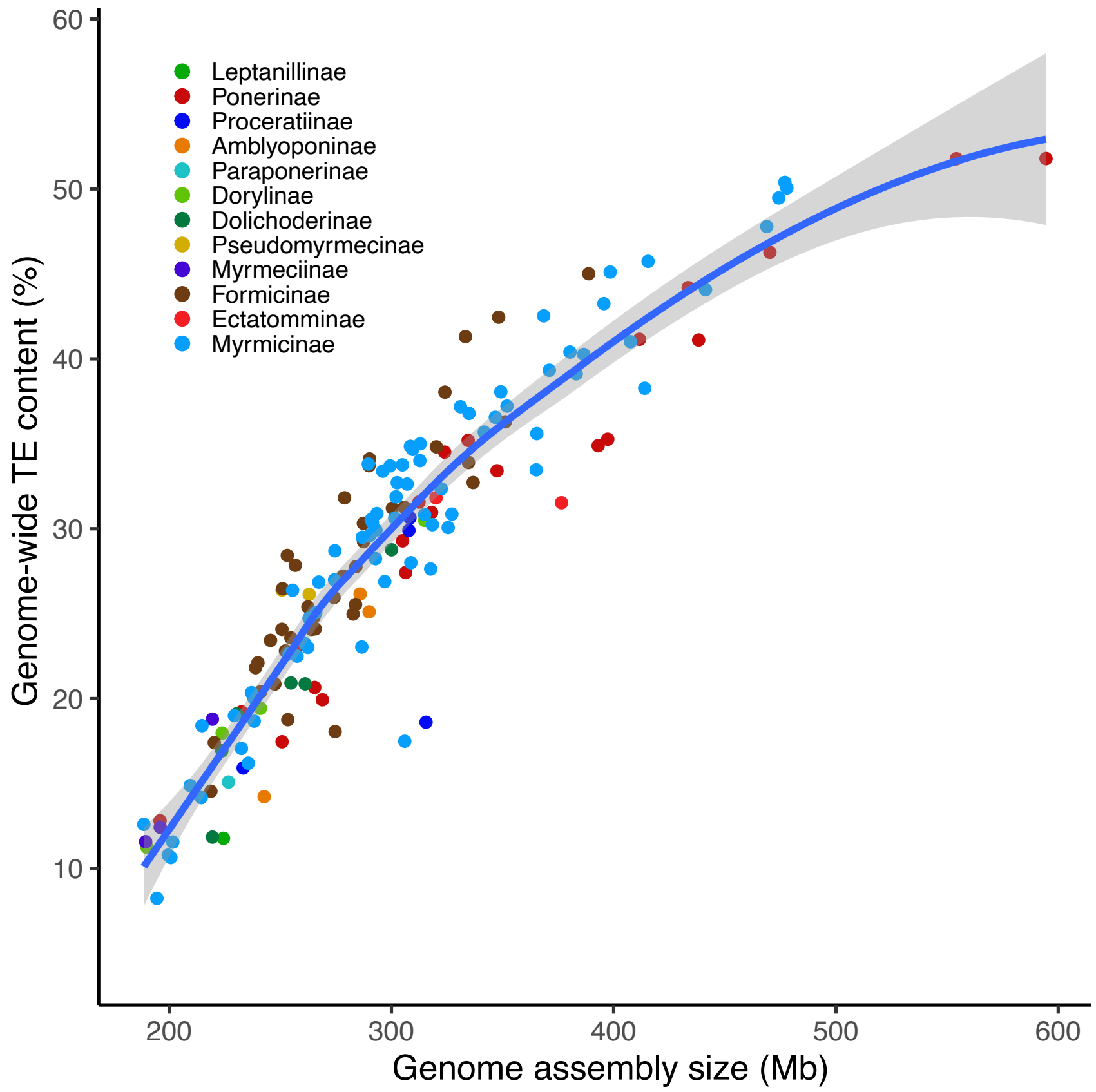

Figure S3: Correlation between genome assembly size (Mb, x-axis) and genome-wide TE content (%, y-axis) for different ant species, colored by ant subfamily. Each dot represents a single genome included in the study. The blue line shows a loess fitted line with `geom_smooth`. Grey shading shows 95% confidence intervals.

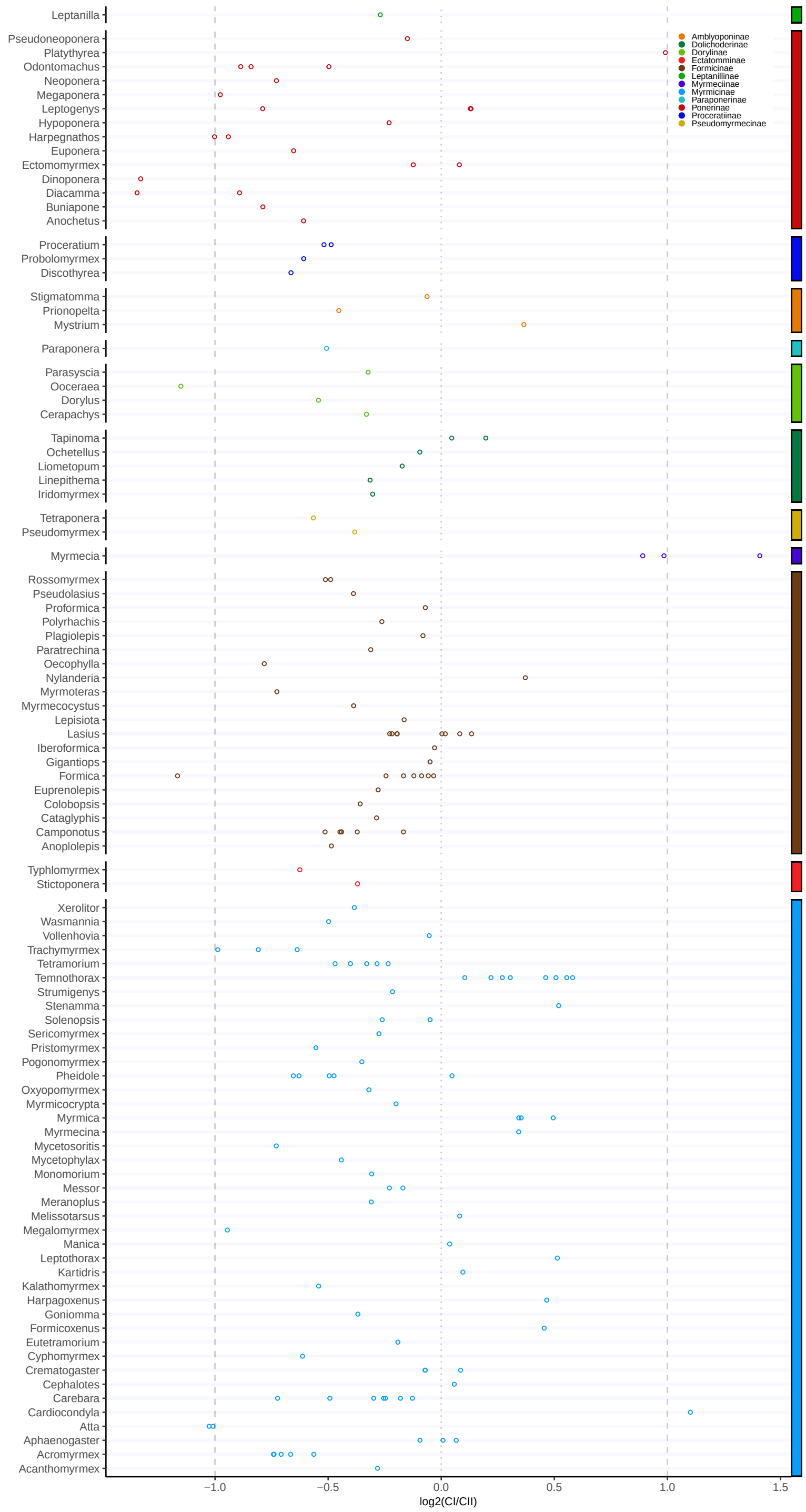

Figure S4: Ratio (log2-transformed) of genome-wide Class 1 and Class 2 TE content in all analyzed ant genomes, grouped by genus. Subfamilies of ants are colored as in Figure S1. The ratio of Class I RNA transposons to Class II DNA transposons varied considerably across genera, even within subfamilies. In *Cardiocondyla*, *Myrmecia*, and *Platythyrea*, retrotransposons were twice as abundant as DNA transposons. Conversely, in *Harpegnathos*, *Megaponera*, *Atta*, *Diacamma*, *Ooceraea*, and *Dinoponera*, DNA TEs were twice as abundant as RNA TEs. These findings are consistent with lineage-specific dynamics, where expansions and contractions of TEs have shaped the repeat landscapes of ant lineages differently.

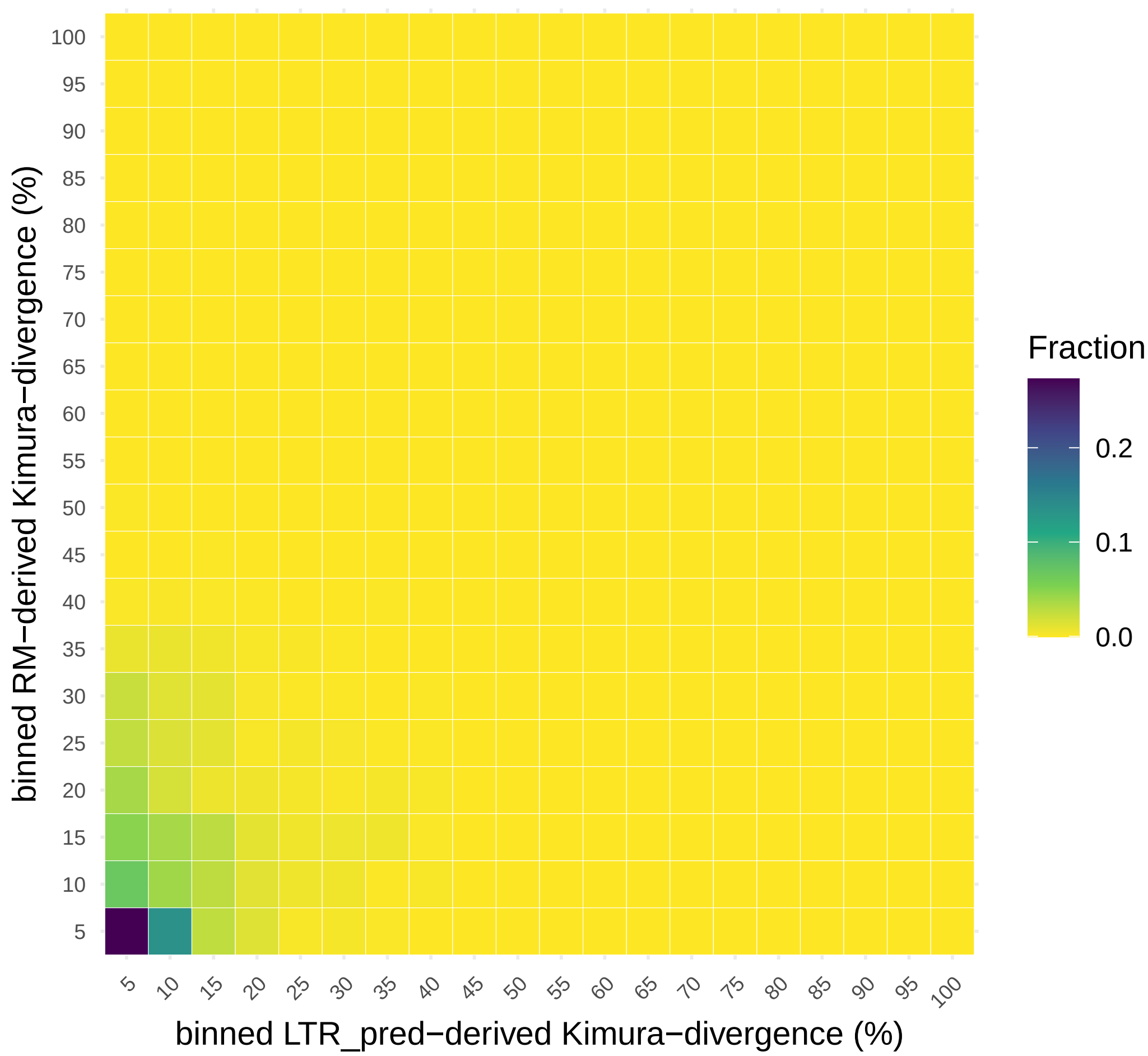

Figure S5: Comparison of Kimura-corrected LTR divergence estimates from structural annotation and `RepeatMasker` across 25 ant genomes. Heatmap showing the correspondence between long terminal repeat divergence estimates derived from `LTRpred` (https://github.com/drostlab/LTRpred) structural annotations (x-axis) and `RepeatMasker` Kimura-corrected divergence values (y-axis), binned in 5% intervals. Each cell represents the fraction of all analyzed insertions falling within that combination of divergence bins (color scale). Only insertions where `LTRpred` annotated a long-terminal repeat, `RepeatMasker` annotated the locus as an LTR element, and `LTRpred` and RepeatMasker start/end coordinates agreed within 5 bp were retained for this comparison. LTR-pred similarity scores were converted to Kimura-corrected distances using a transition/transversion ratio of 2.0 prior to binning. Concentration of counts at low divergence values from both estimators indicates broad concordance between the two methods for recently inserted elements. Species included: *Acromyrmex echinatior*, *Aphaenogaster exasperata*, *Cardiocondyla obscurior*, *Cerapachys sulcinodis*, *Crematogaster* cf. *rogenhoferi*, *Diacamma* cf. *indicum*, *Ectomomyrmex* cf. *javanus*, *Ectomomyrmex javanus*, *Formica cinerea*, *Harpagoxenus sublaevis*, *Harpegnathos venator*, *Lasius flavus* s1, *Lasius niger*, *Leptogenys kitteli*, *Megaponera analis*, *Messor barbarus*, *Myrmica angulata*, *Myrmica rubra*, *Mystrium camillae*, *Platythyrea punctata*, *Solenopsis invicta*, *Stenamma debile*, *Temnothorax ravouxi*, *Temnothorax rugatulus*, *Tetraponera rufonigra*.

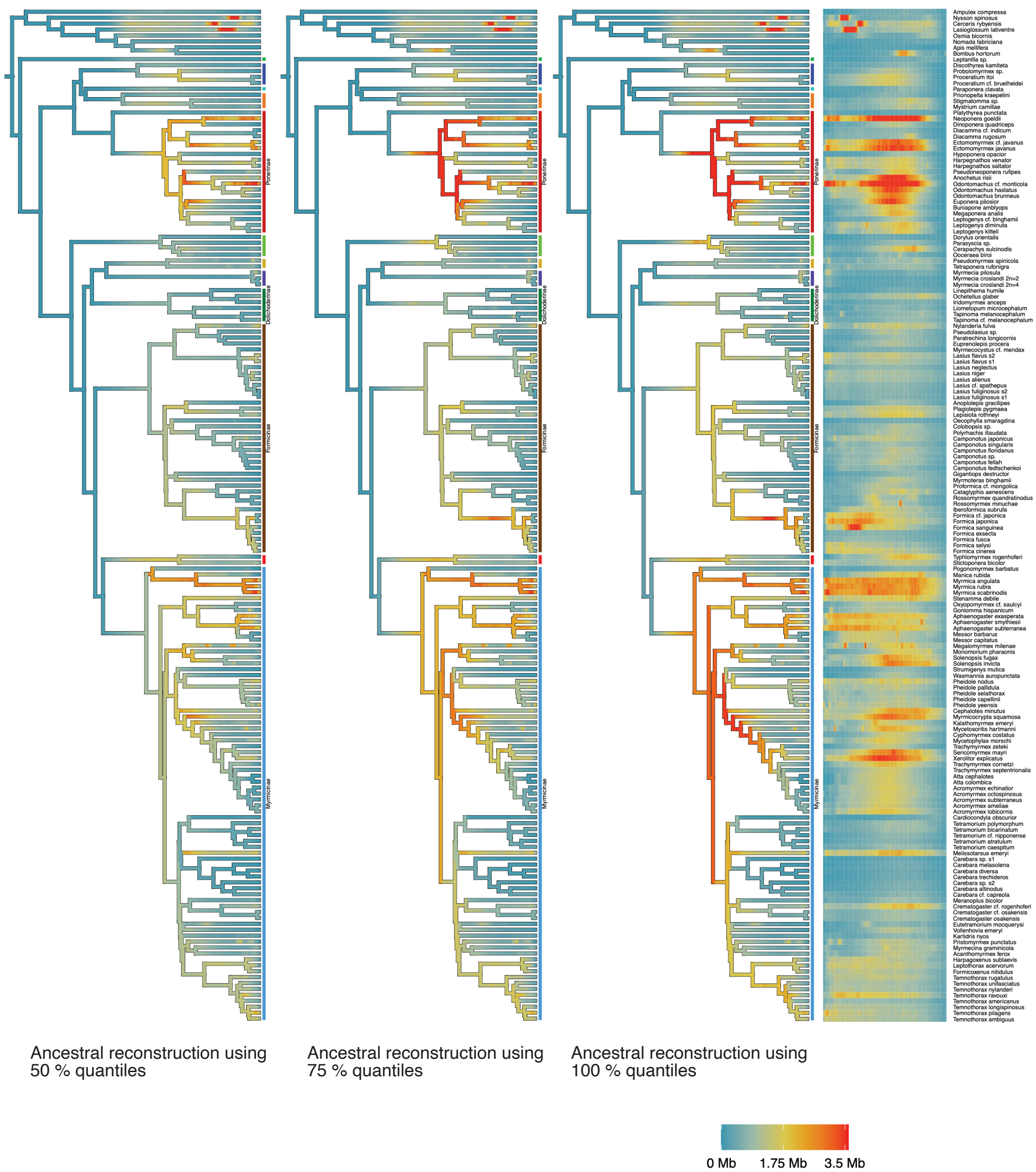

Figure S6: The heatmap on the right shows modeled insertion times of TEs according to Kimura-distance-based copy divergence in ants and eight outgroup species over the last 100 My. Periods of intensified TE proliferation are characterized by increased acquisition of TE sequences and appear in red (up to 3.5 Mb per 1 My). Periods of reduced TE proliferation are shown in blue. One My segments on branches in the phylogeny are colored according to estimated gains of TEs based on ancestral state reconstructions using 50%, 75% or 100% quantiles.

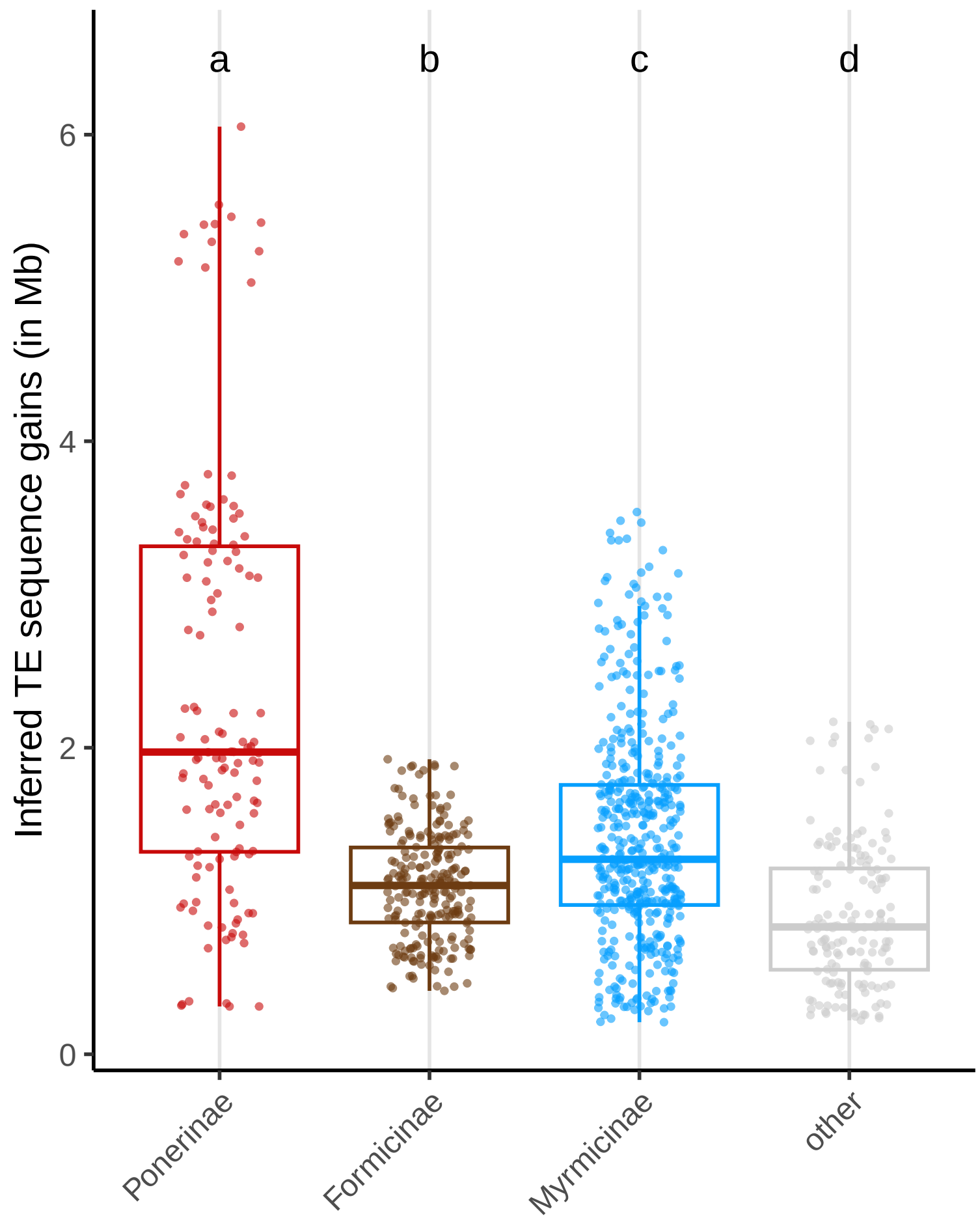

Figure S7: Inferred TE sequence gains across species belonging to the subfamilies Ponerinae, Formicinae, Myrmicinae, and the remaining nine subfamilies pooled during the reconstructed TE proliferation peak (see Fig. 2B). Letters indicate significant differences based on Dunn's test (see Supplementary Material S1.2).

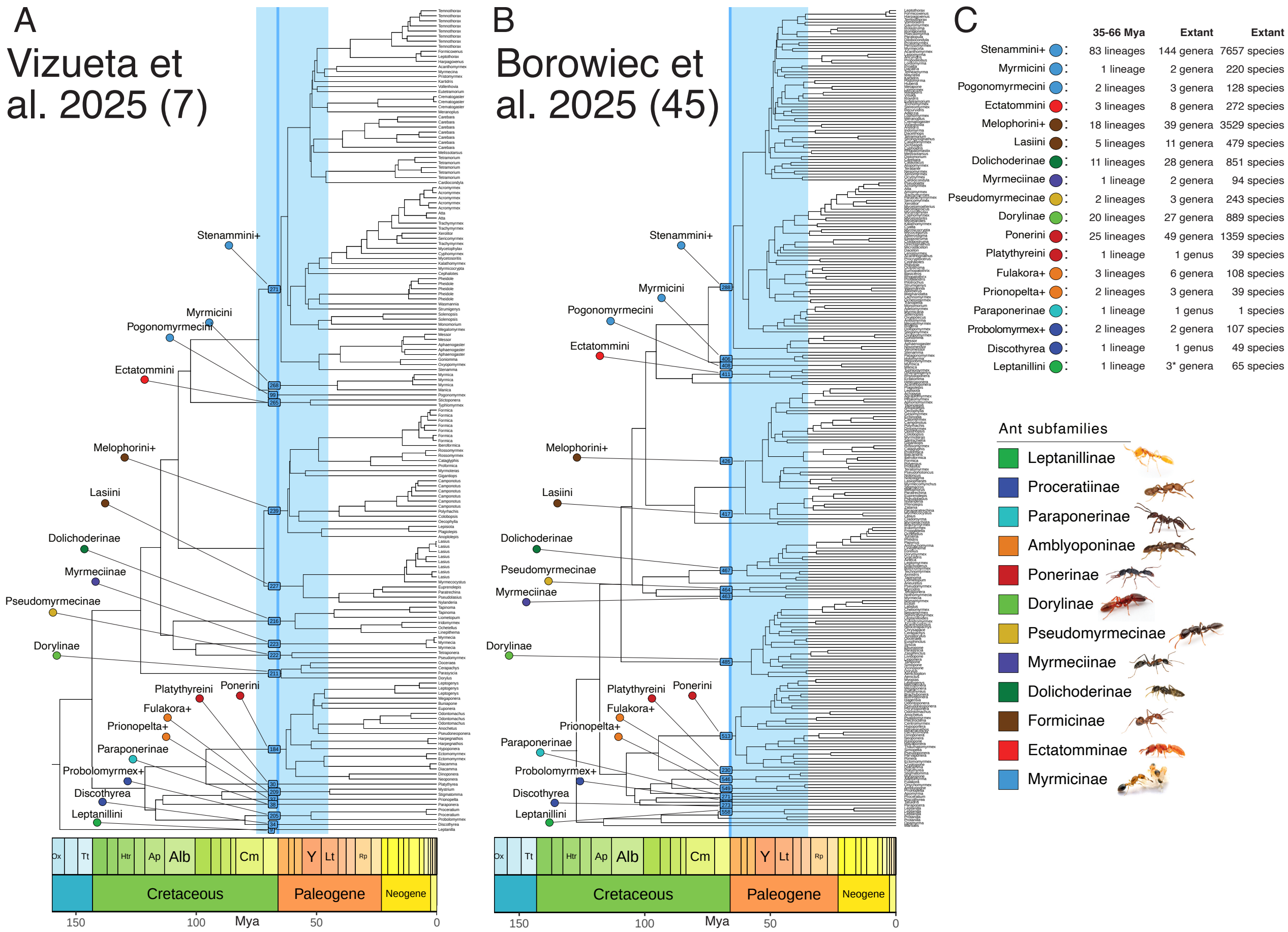

Figure S8: Time-calibrated phylogenetic trees of ants and diversification metrics for Cretaceous-Paleogene lineages. Time-calibrated phylogenetic trees based on (A) Vizueta et al. 2025 (7) and (B) Borowiec et al. 2025 (45). Branches that existed at 66 Mya according to the Vizueta et al. phylogeny are highlighted and labeled in panel A, with corresponding branches highlighted in the Borowiec et al. UCE-based phylogeny shown in panel B. Branch labels indicate the extant ant subfamilies, tribes, or genera that are descendants of each respective lineage. Panel C quantifies the number of lineages that emerged from each highlighted branch between 66 and 35 Mya (based on the UCE phylogeny from panel B), along with the number of described extant genera and species for each lineage. *: Borowiec et al. 2025 (45) included three Leptanillini genera (*Leptanilla*, *Noonilla* and *Yavnella*) that were recently synonymized to *Leptanilla*.

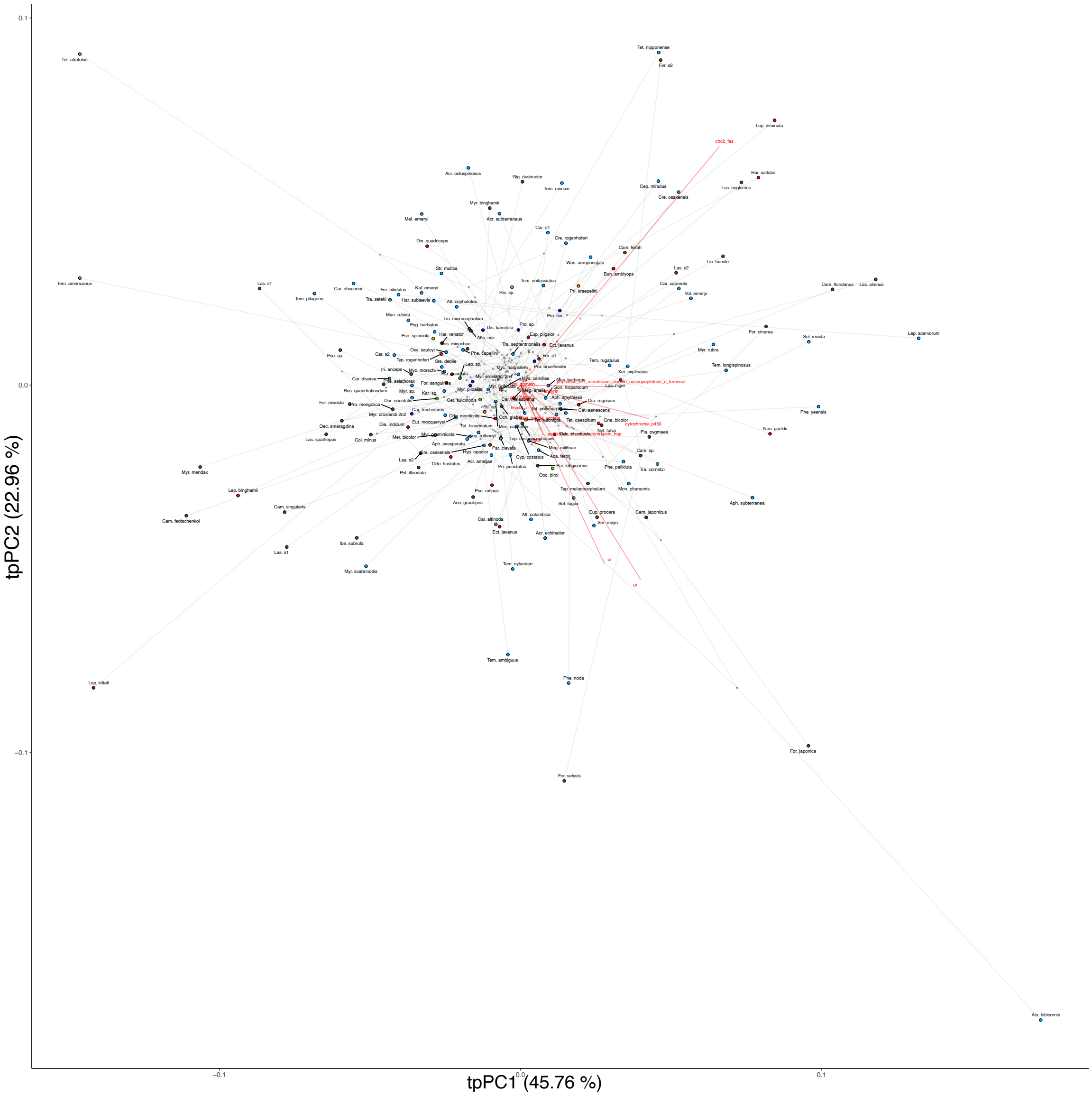

Figure S9: Principal components (PC) 1 (x-axis) and 2 (y-axis) produced by transformed phylogenetic principal component analysis (tpPCA) of gene family sizes of the 12 largest gene families. Each dot (colored by ant subfamily) represents the principal component scores for an included taxon. Internal nodes are illustrated as open circles, with phylogenetic relationships indicated by grey lines. Loadings on PC1 and PC2 are shown as red arrows for each included gene family. Abbreviated species names are given along each colored dot.

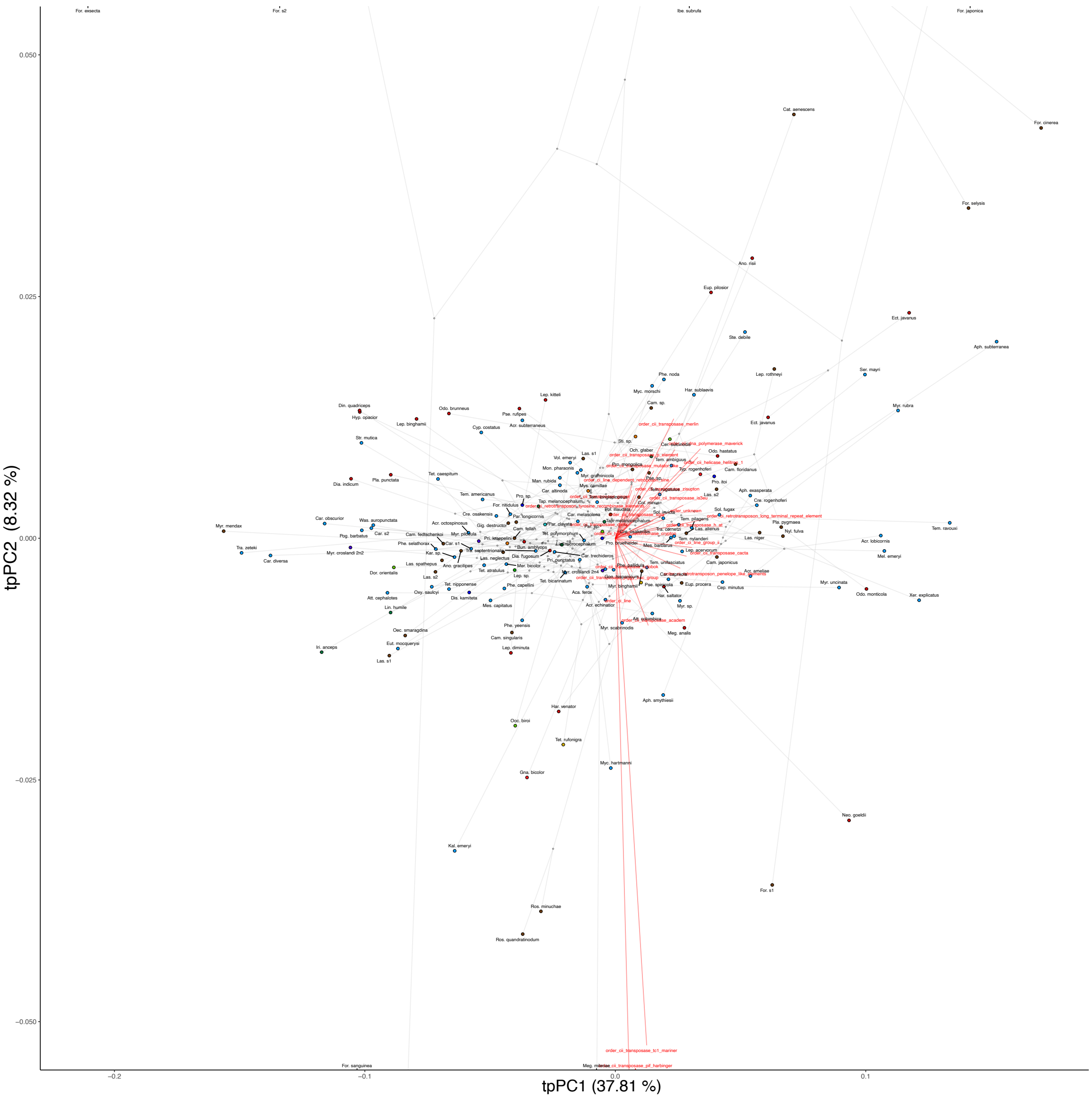

Figure S10: Principal components (PC) 1 (x-axis) and 2 (y-axis) produced by transformed phylogenetic principal component analysis (tpPCA) of genome-wide TE content of the 24 most abundant TE orders. Each dot (colored by ant subfamily) represents the principal component scores for an included taxon. Internal nodes are illustrated as open circles, with phylogenetic relationships indicated by grey lines. Loadings on PC1 and PC2 are shown as red arrows for each included TE order. Abbreviated species names are given along each colored dot.

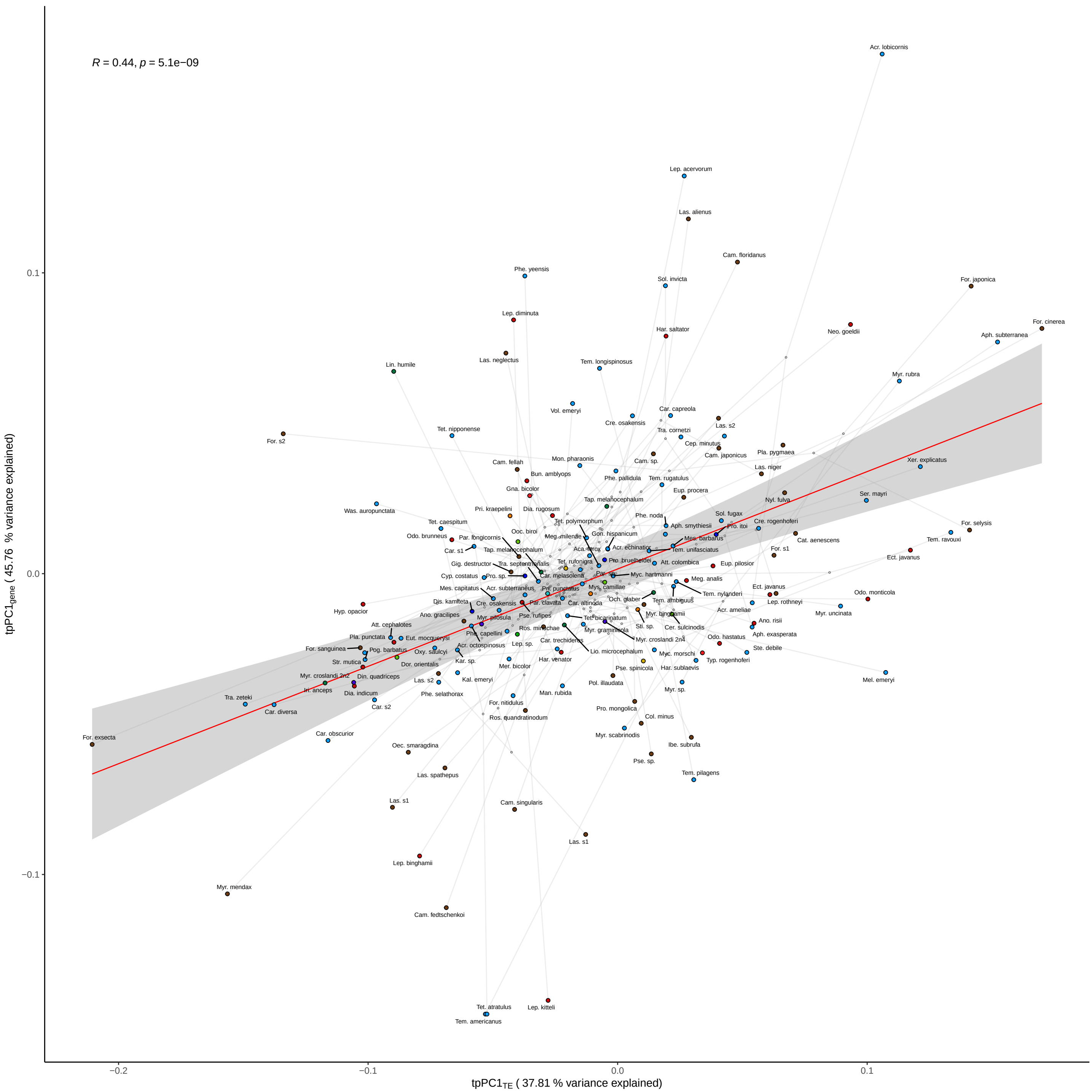

Figure S11: Correlation of (phylogenetically corrected) $tpPC1_{TE}$ (x-axis) and $tpPC1_{gene}$ (y-axis) as shown in Figure 3C with abbreviated species names along each colored dot.

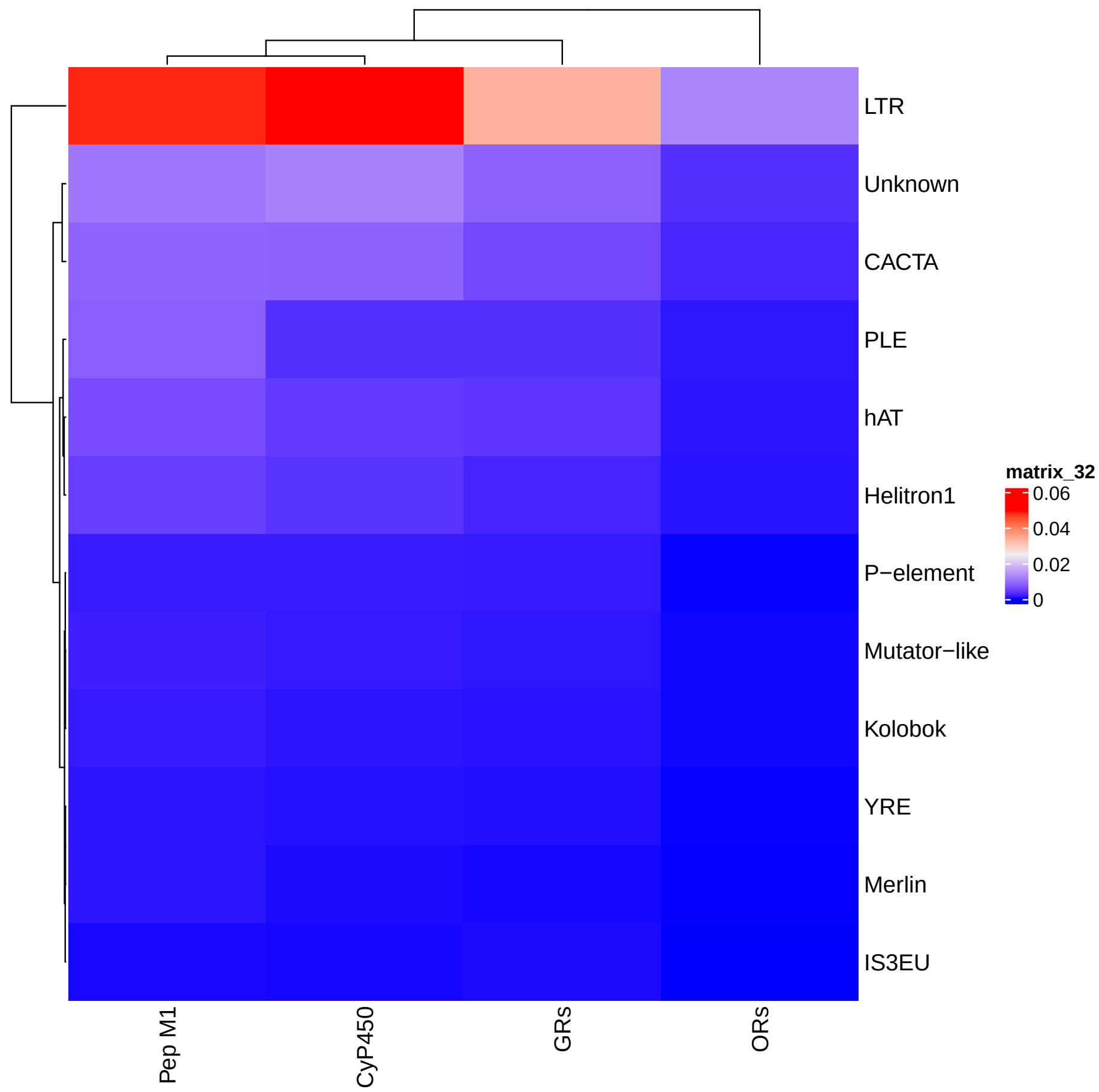

Figure S12: Heatmap showing the slope (cell color) of correlations for GR, OR, PepM1, and CyP450 gene family sizes (columns) and genome-wide content (%) of different TE orders (rows). Note that the slope is positive for each comparison.

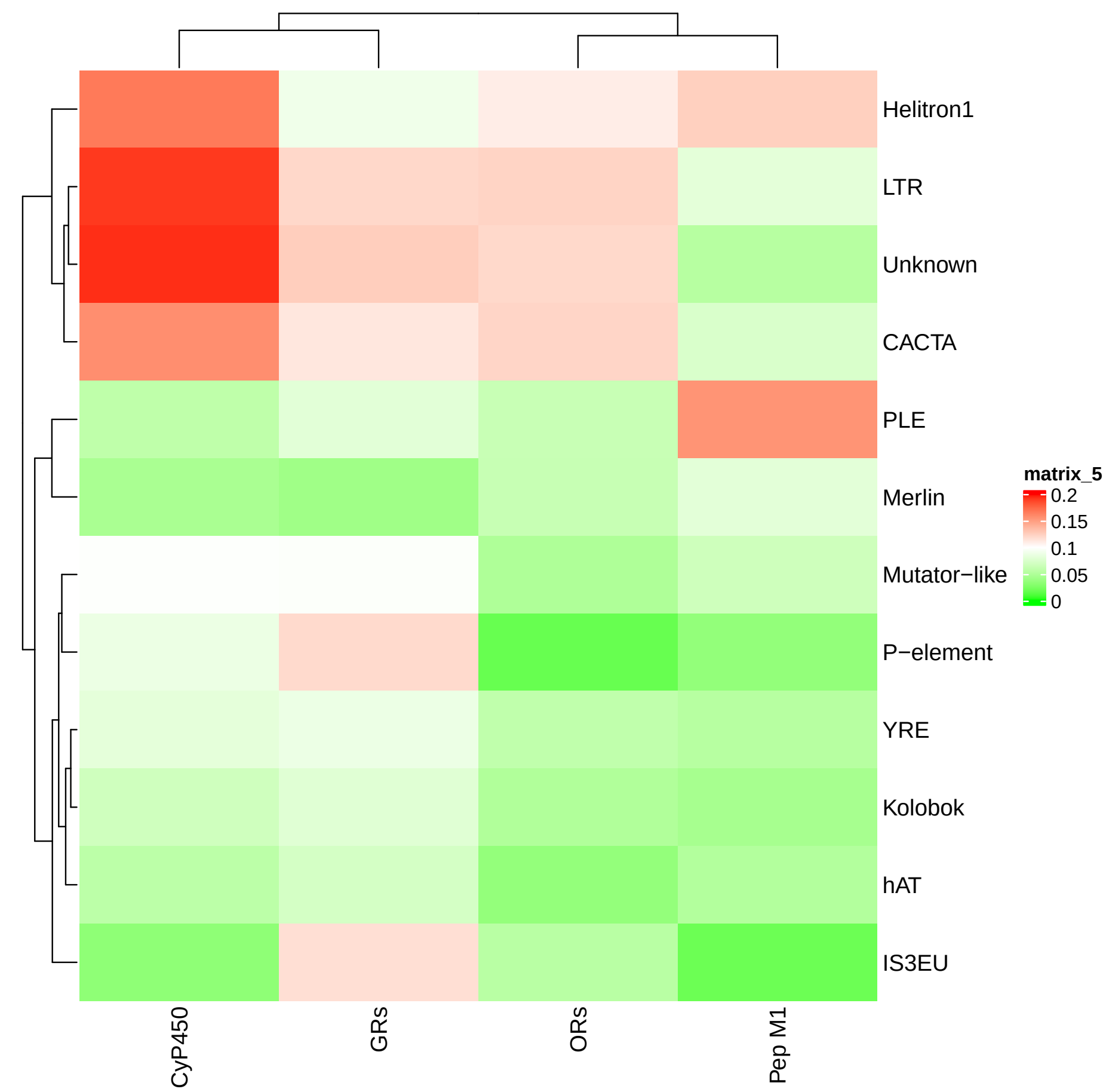

Figure S13: Heatmap showing the adjusted R-squared (cell color) correlation coefficients for the key gene families (GRs, ORs, PepM1, and CyP450 columns) and genome-wide content (%) of different TE orders (rows).

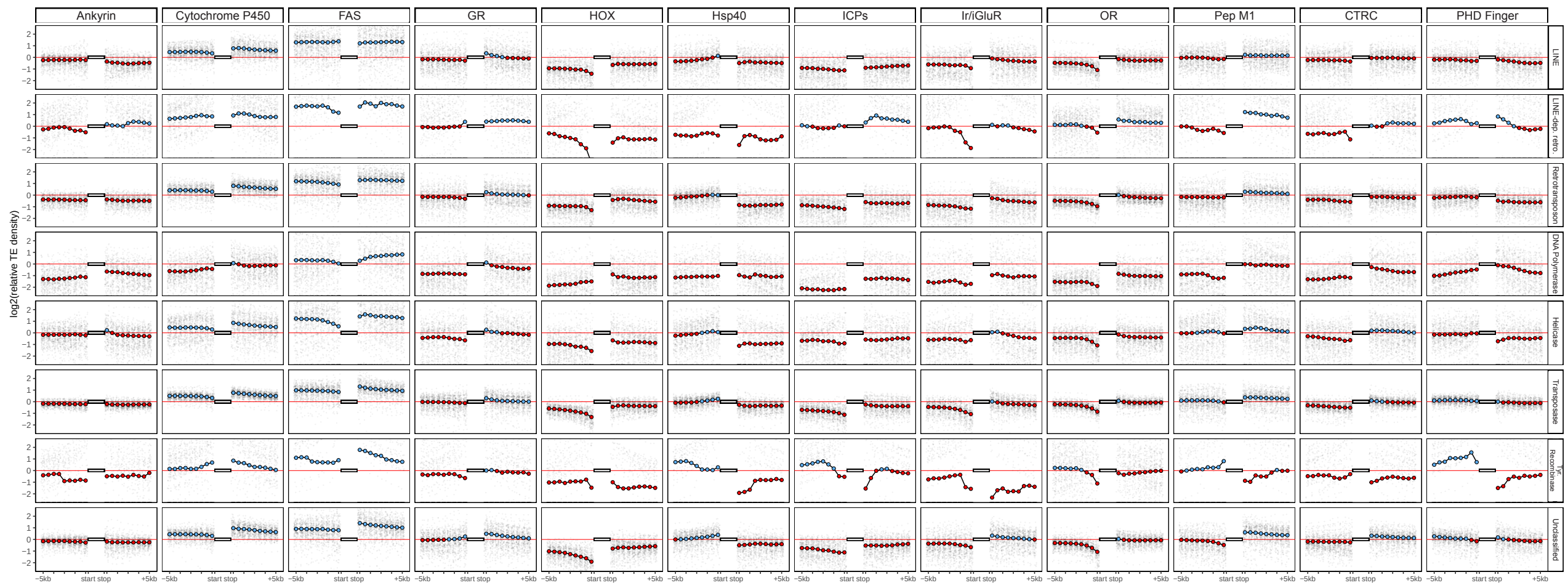

Figure S14: Normalized TE densities in different genomic windows relative to genes from different gene families across 163 ant genomes. Filled points represent average, normalized TE densities for the respective gene families and TE orders, at intervals of 500 bp for 4.5 kb up- and downstream of genes. Relative TE densities are calculated as log2-transformed ratios between the sum of TE-occupied bases in a certain window relative to the overall TE density for all other genes.

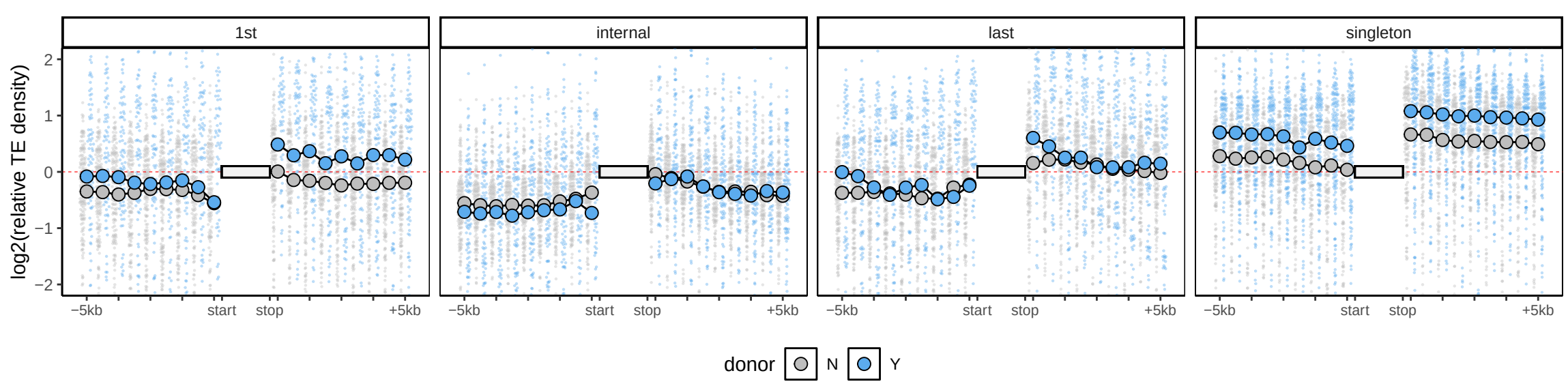

Figure S15: Normalized TE density analysis across 163 ant genomes for GRs, separated by location of GR genes within tandem arrays. GR genes in tandem arrays have been categorized as the first gene of an array (1st), an internally located gene (internal), the last gene of an array (last), or a singleton GR gene (with a distance of at least 10,000 bp to any other GR gene). Blue points represent inferred donor genes (i.e. direct paralogs) for displaced duplicates, while grey-filled points represent non-donor genes. TE density is substantially higher around internally located donor GRs compared to non-donor genes, similar to patterns observed for ORs.

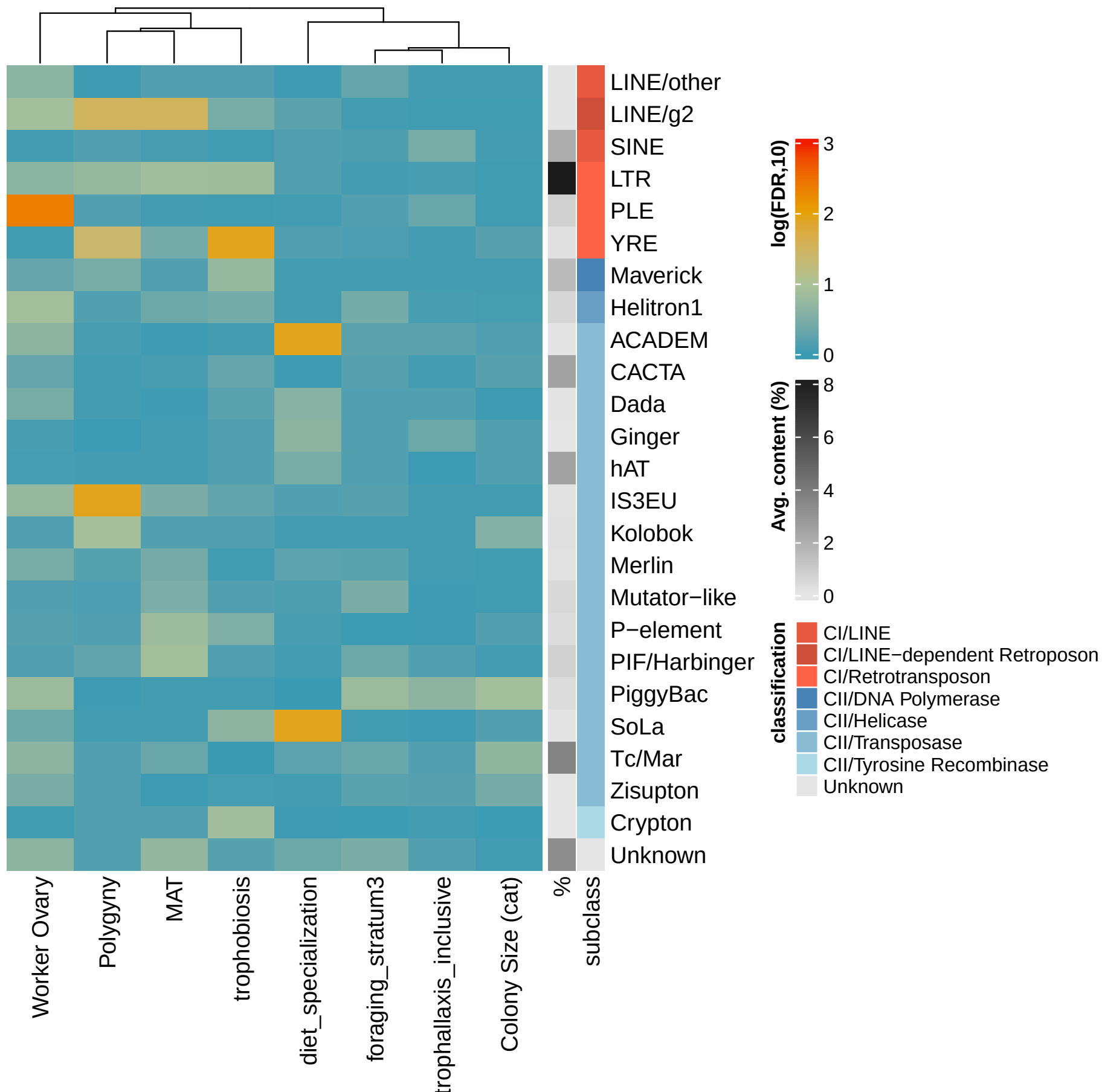

Figure S16: Heatmap showing log10-transformed p-values of pgls models correlating genome-wide abundances of different TE orders (rows) with different ecological or life-history traits (columns). Across all tested traits and TEs, we found significant correlations ($fdr < 0.05$) for *line_group_ii∼polygyny* (0.0316), *retrotransposon_tyrosine_recombinase_elements∼polygyny* (0.0421), *transposase_is3eu∼polygyny* (0.0116), *retrotransposon_penelope_like_elements∼worker_ovary* (0.0044), *line_group_ii∼mat_mean* (0.0316), *transposase_academ∼diet_specialization* (0.0116), *transposase_sola∼diet_specialization* (0.0116), and *retrotransposon_tyrosine_recombinase_elements ∼trophobiosis* (0.0116).

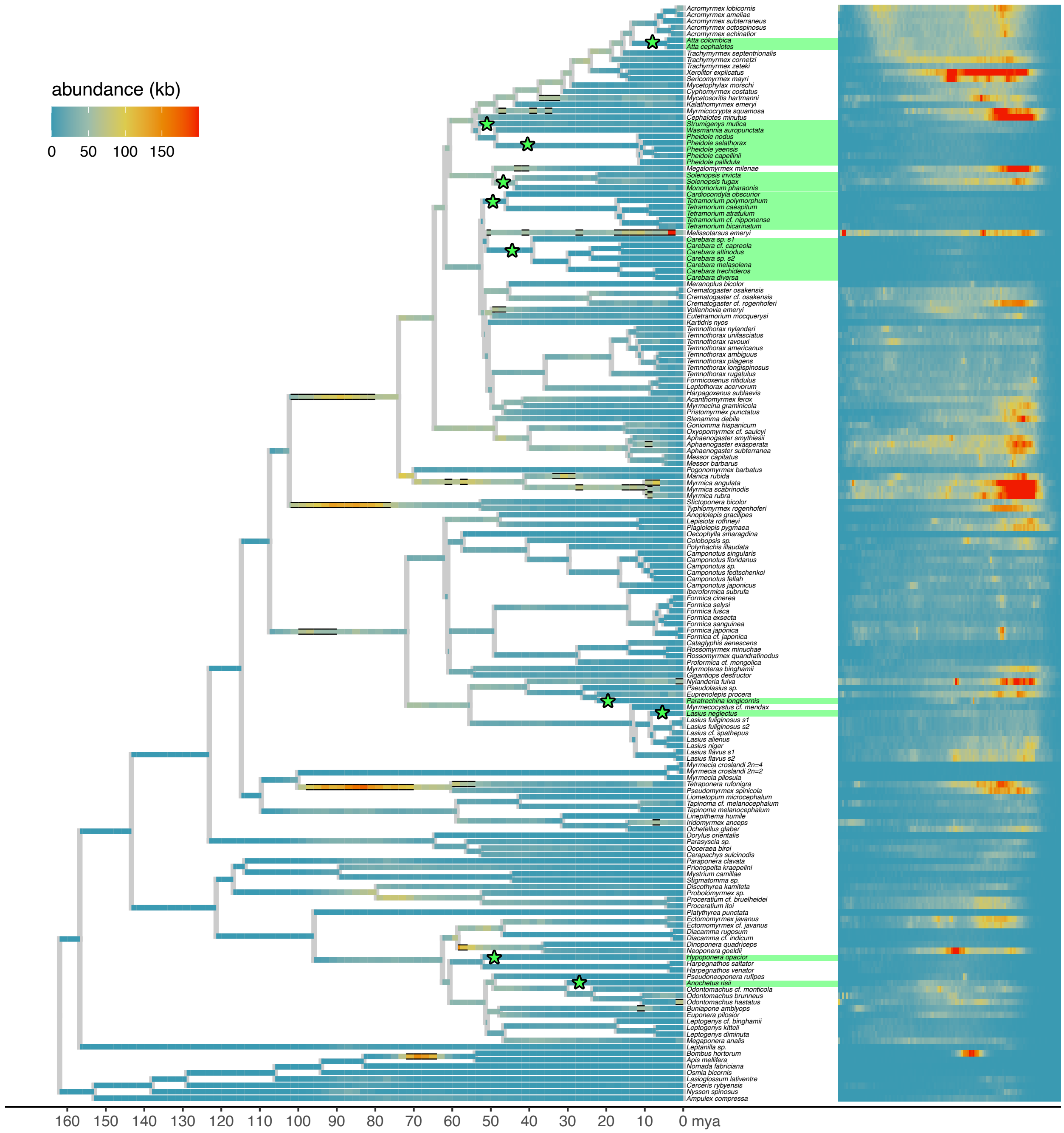

Figure S17: The heatmap on the right shows modeled insertion times of Penelope-like elements (PLE) according to Kimura-distance-based copy divergence in ants and eight outgroup species over the last 100 My. Green stars mapped onto the phylogeny on the left show evolutionary transitions towards workers without ovaries (Vizueta et al. 2025) and green-shaded backgrounds highlight species where workers have lost ovaries. The heatmap on the right shows the same data, but colored by the amount of Penelope-like element sequence gained per genome per My. Consistent with the pgls model, there is a clear reduction of PLEs in several of the genera with inferred losses of worker ovaries, in particular in the Myrmicine genera *Carebara*, *Pheidole*, *Cardiocondyla*, and *Tetramorium*.

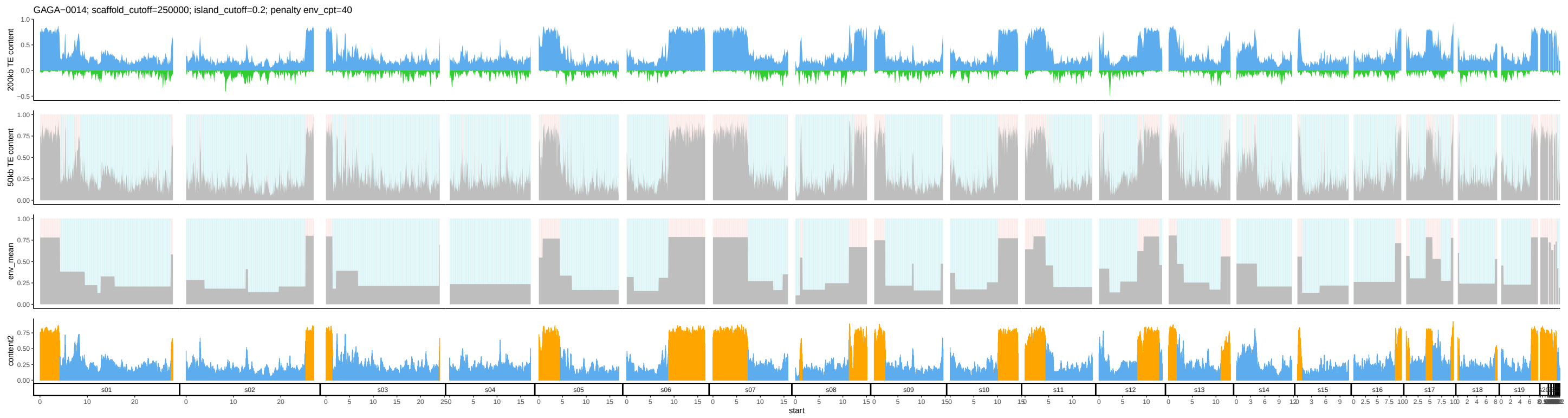

Figure S18: Example of genome compartmentalization quantification using sliding window analyses and changepoint modelling. The figure shows the analysis of genome assembly GAGA-0014 (*Acromyrmex echinatior*). The top panel represents TE content (in blue) and exon content (in green, y-axis) along scaffolds (x-axis) in 200 kb sliding windows. The second panel shows TE content in 50 kb windows, with regions with high TE content (>50%) highlighted with a pink background. The third panel shows the mean changepoint model of TE content applied to the 200 kb sliding windows. Annotated TE islands as continuous regions had considerably more TEs than the genomic background, as shown in orange in the bottom panel.

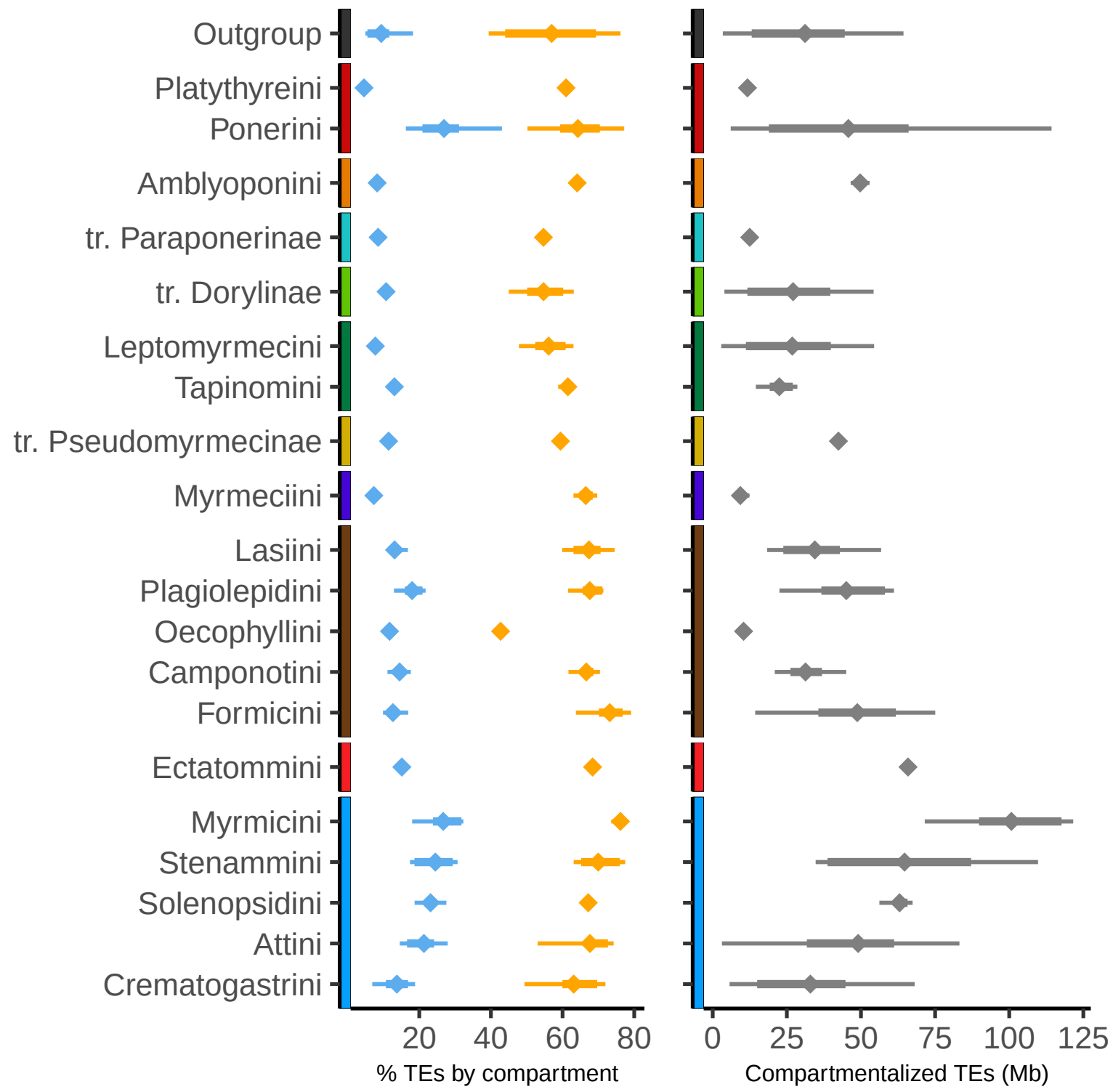

Figure S19: Transposable element (TE) compartmentalization varies across ant tribes. The left panel shows differences in average TE content (%) between TE islands (orange) and TE-poor genomic regions (blue) across ant tribes, organized by subfamily (vertical bars using color coding from Figure 4). The right panel displays total TE content within TE islands (Mb) for each tribe. Filled diamonds represent means, thick lines show 25th and 75th percentiles, and thin lines indicate 5th to 95th percentiles. TE islands contain 67% TE sequences on average, compared to 15% in other genomic regions. Phylogenetic patterns emerged despite high within-tribe variation, as the Ponerini, Attini, Solenopsidini, Stenammini, and Myrmicini show relatively high TE content outside islands, whereas Platythyreini, Amblyoponini, and Formicini exhibit stronger compartmentalization. TE island content varies dramatically, exceeding 100 Mb in six species (*Neoponera goeldii*, *Myrmica scabrinodis*, *M. rubra*, *Stenamma debile*, *Aphaenogaster subterranea*, *Ectomomyrmex javanus*) but remaining below 10 Mb in most *Carebara* species. This variation suggests that lineage-specific factors drove TE island evolution in the past.

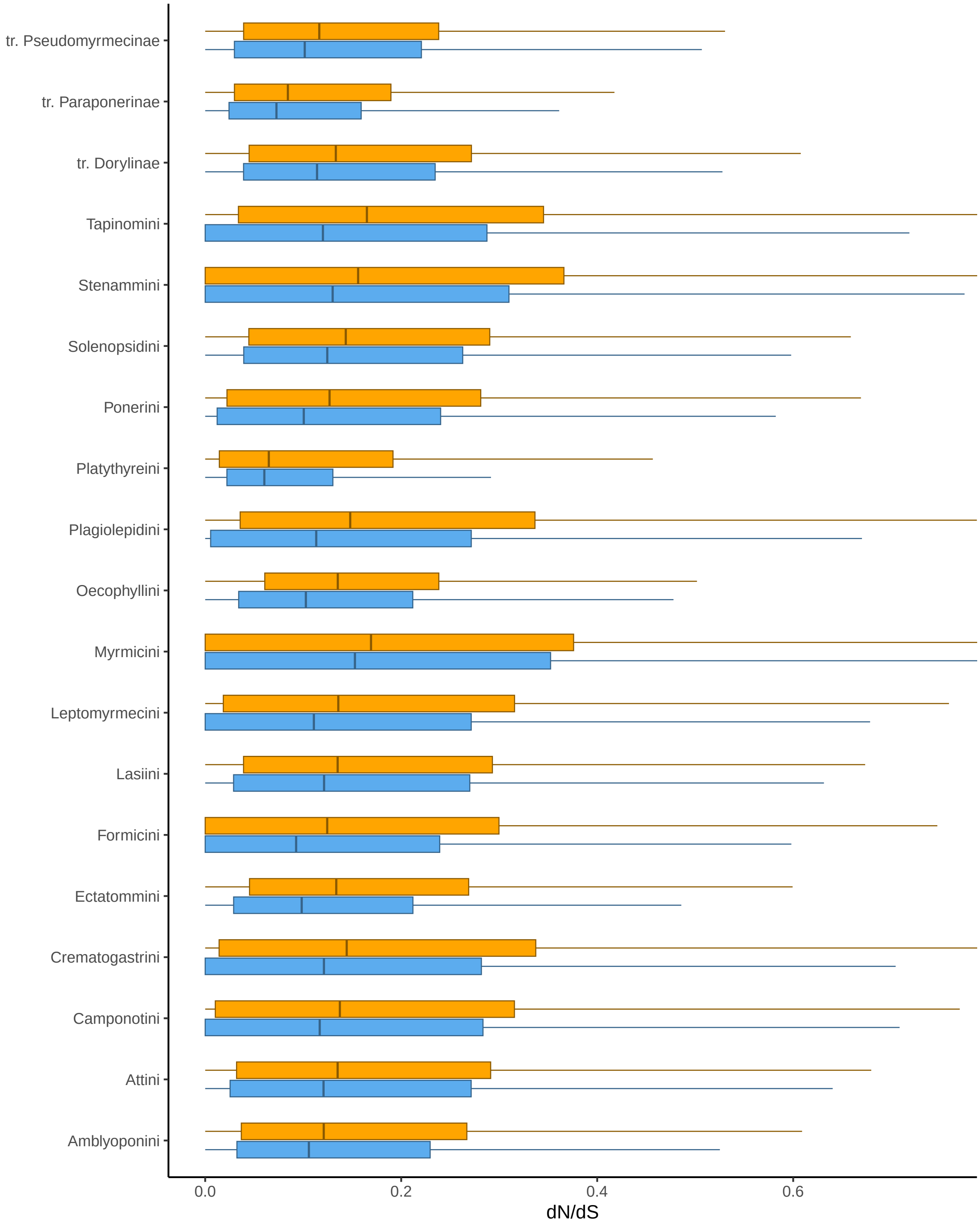

Figure S20: Increased evolutionary rates in TE island genes. Boxplots show median, interquartile ranges (IQR, boxes), and 1.5× IQR (whiskers) of background evolutionary rates for genes located in TE islands (orange) or TE-poor (blue) genomic regions across the different ant tribes along the y-axis.

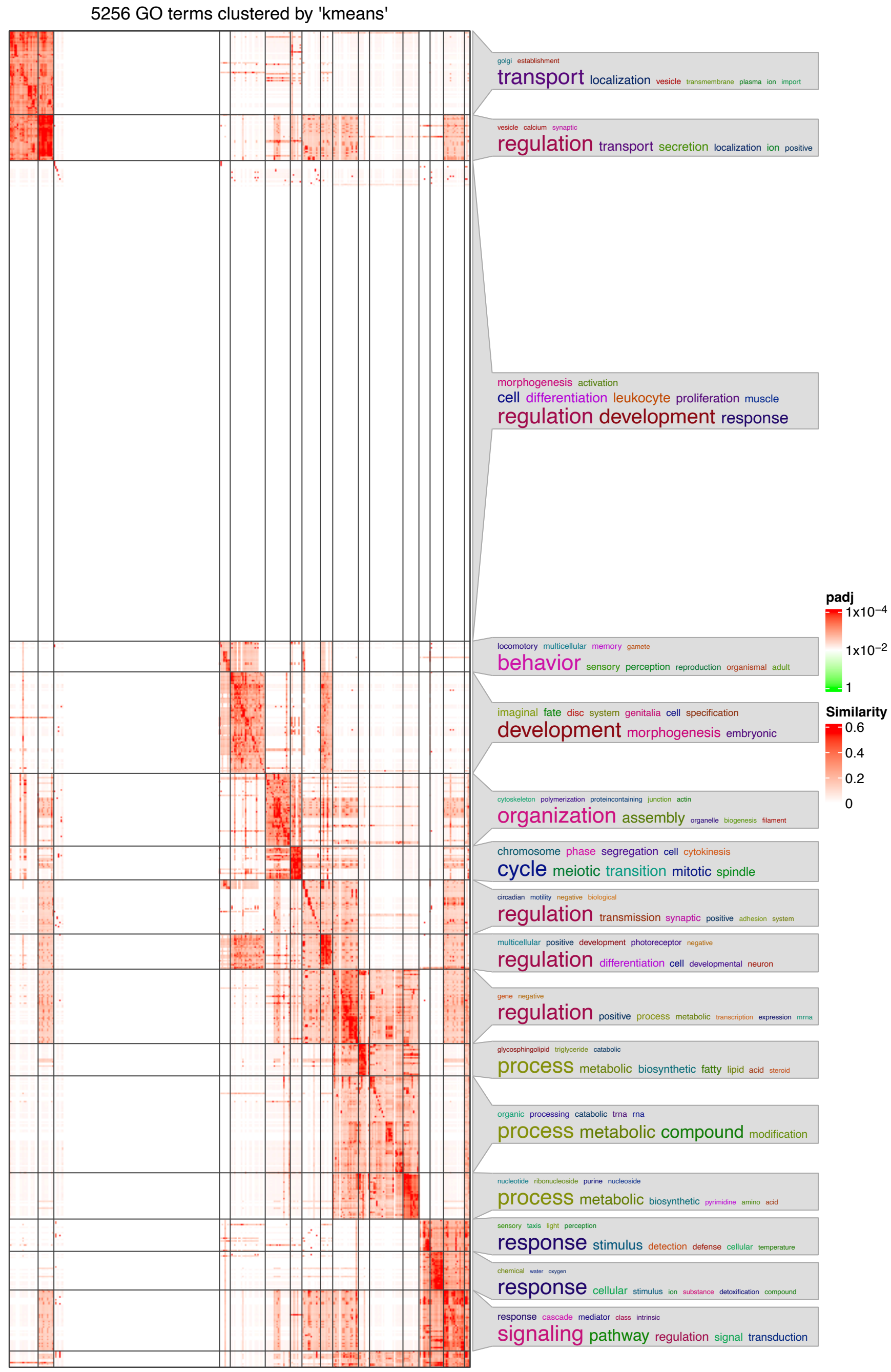

Figure S21: Overview of the major biological themes enriched among genes in TE islands of ant genomes. Clustering of enriched Gene Ontology (GO) terms using the `simplifyEnrichment` package in `R` (with `simplifyEnrichmentFromMultipleLists()`, `p-value cutoff = 0.001, clustering method = "kmeans"` using `org.Dm.eg.db_3.18.0`). The left panel shows heatmaps of pairwise semantic similarities between significant GO terms, where functionally related terms are grouped into clusters by the *binary cut* algorithm. The right panel displays word clouds summarizing the representative biological concepts in each cluster, with word size being proportional to term frequency among cluster members.

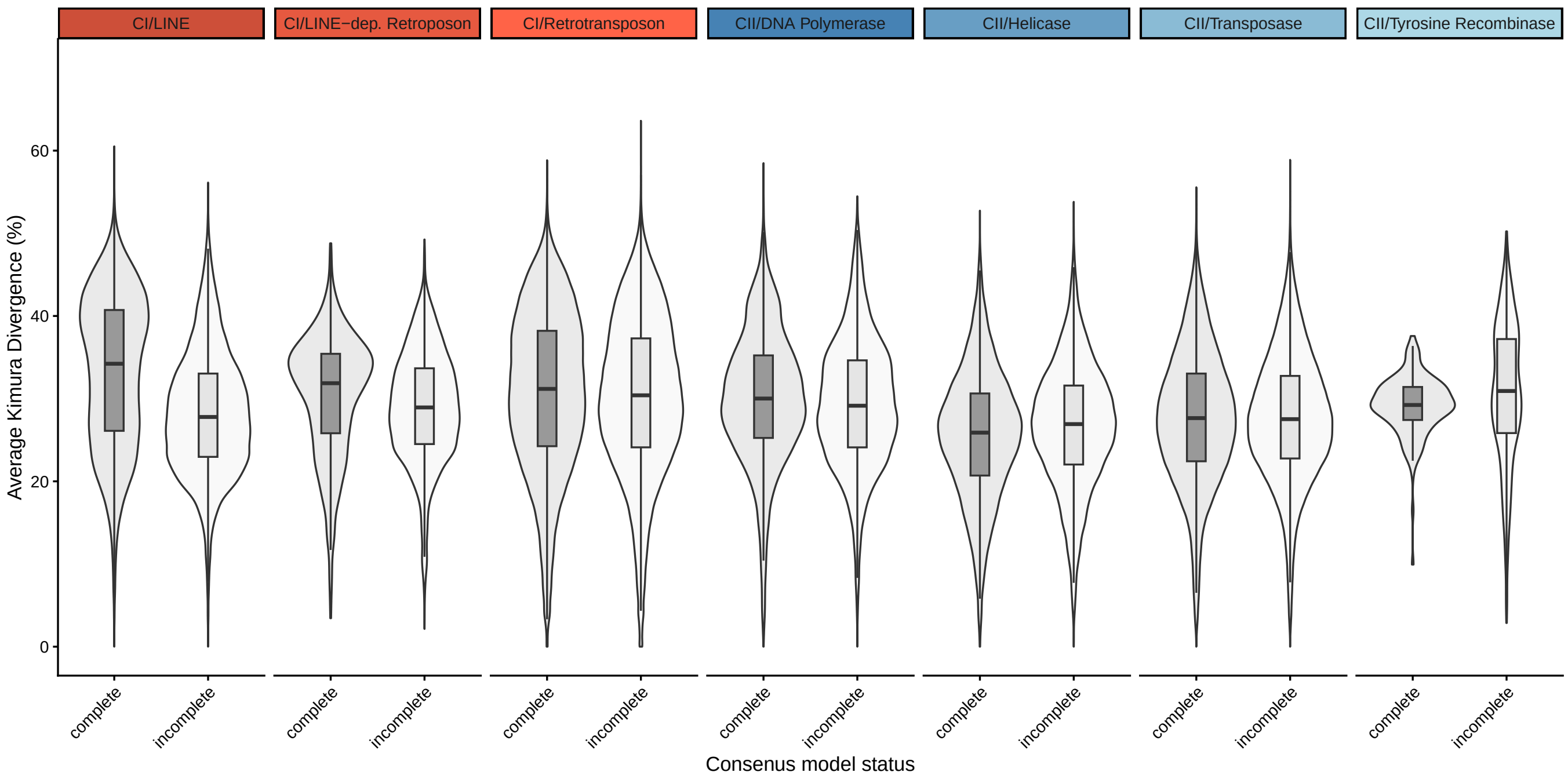

Figure S22: Kimura distances of annotated TE copies to their respective consensus sequences for different TE orders (x-axis), comparing consensus models annotated as "complete" or "incomplete" by `MCHelper`. For four TE orders (CI/LINE, CI/LINE-dep. Retroposons, CI/Retrotransposons, CII/DNA Polymerase), average Kimura distances were higher for "complete" than "incomplete" consensus models, while for the other three orders (CII/Helicase, CII/Transposase, CII/Tyrosine Recombinase) we observed the opposite pattern.

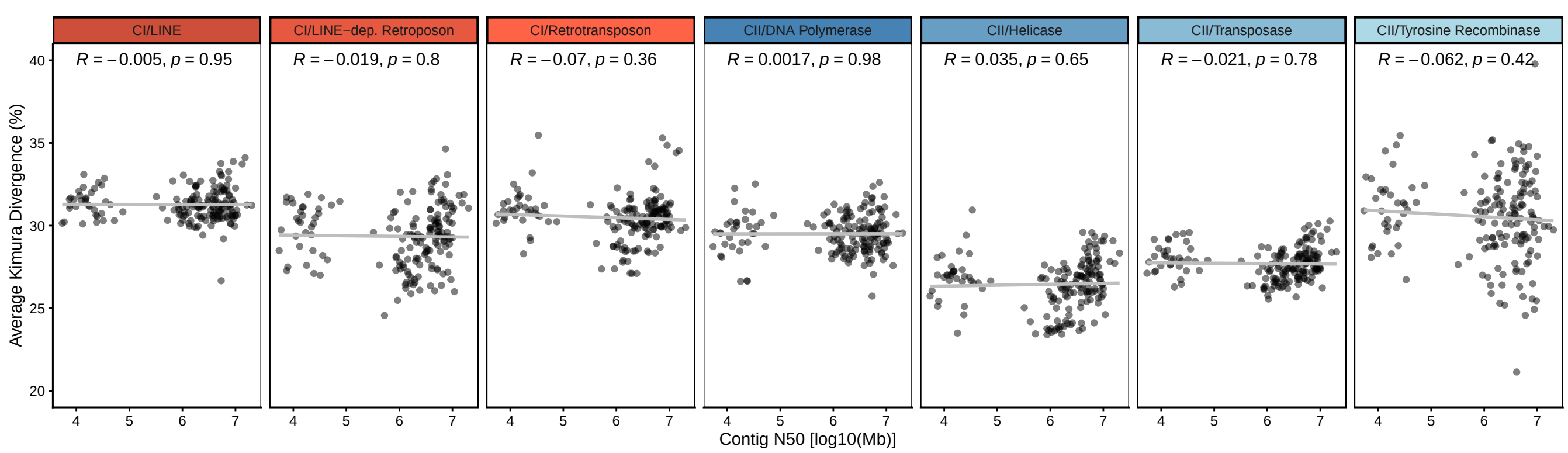

Figure S23: Impact of genome contiguity (log10(N50 contig), x-axis) on average Kimura divergence estimates (y-axis) across all analyzed genomes. Pearson correlation coefficients (R) and associated p-values are given at the top of each panel. We found no significant correlation for any TE order, suggesting that differences in assembly quality did not bias our estimates of TE insertion dynamics across species.

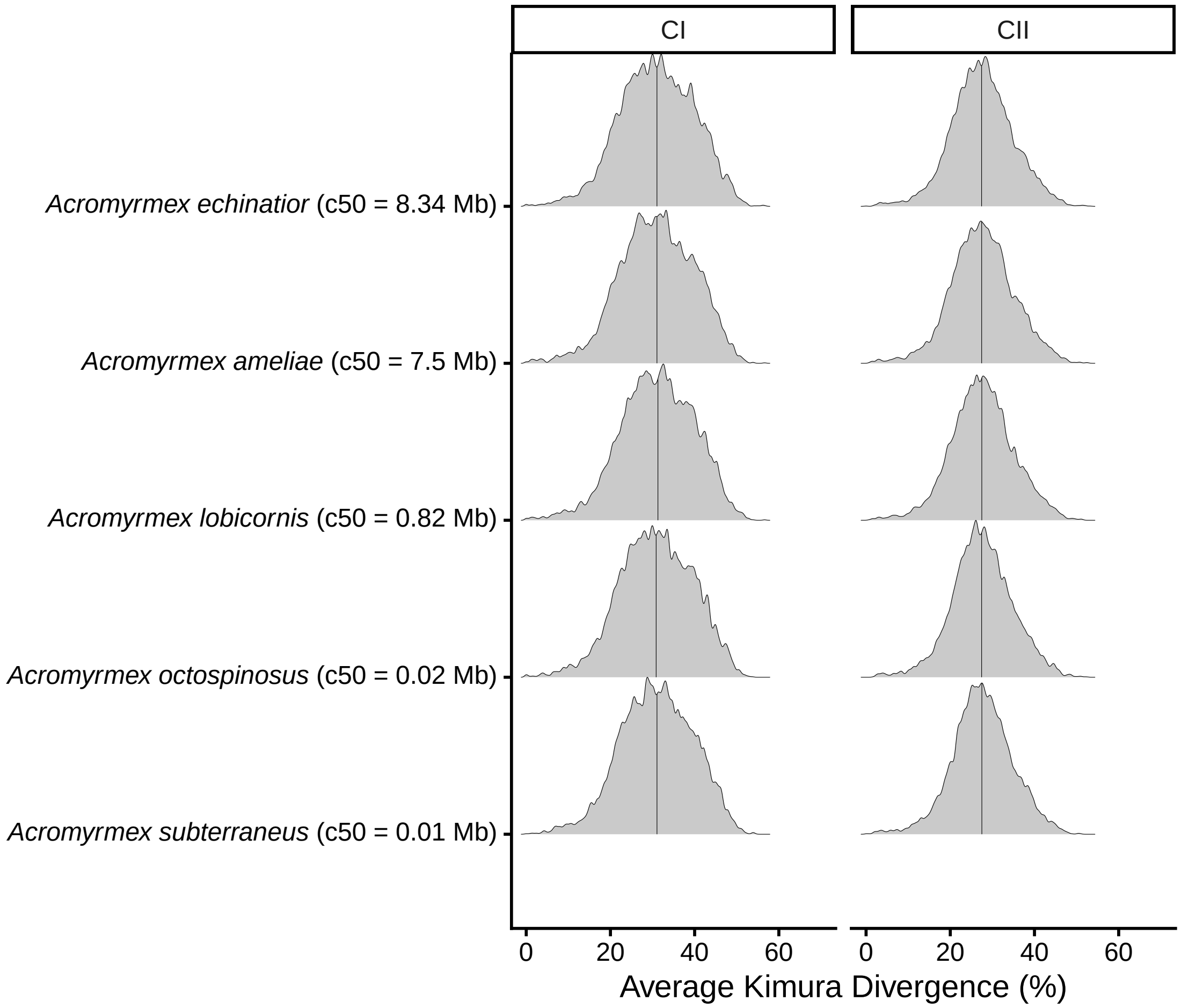

Figure S24: Kimura divergence landscapes for Class 1 (left) and Class 2 TEs (right) across different *Acromyrmex* genomes with varying genome contiguity (contig N50, given in parentheses next to the species name). Vertical lines indicate median Kimura divergences.

S3. Supplementary Tables

Table S1a:	Summary of annotated bases as either Class 1, Class 2 or Unknown in 163 ant genomes and 8 Apoidea outgroup genomes
Table S1b:	Summary of annotated bases grouped by different subclasses of TEs in 163 ant genomes and 8 Apoidea outgroup genomes
Table S1c:	Summary of annotated bases grouped by different orders of TEs in 163 ant genomes and 8 Apoidea outgroup genomes
Table S1d:	Summary of annotated bases grouped by different superfamilies of TEs in 163 ant genomes and 8 Apoidea outgroup genomes
Table S1e:	Summary of annotated bases grouped by different families of TEs in 163 ant genomes and 8 Apoidea outgroup genomes
Table S1f:	Summary of annotated bases grouped by all available levels of classifications of TEs in 163 ant genomes and 8 Apoidea outgroup genomes
Table S1g:	Summary of total annotated repeat bases in 163 ant genomes and 8 Apoidea outgroup genomes

Table S1: The table sheets `a` to `g` are provided in a supplementary file online, with details regarding the total annotated TE content. Each table shows the number of annotated bases for different levels of TE classification.

**Species**	**Description**	**Photographer**	**Figures**
Leptanilla sp.	*Leptanilla* worker cleaning her antennae. Kibale forest, Uganda	Alex Wild	1,2
Proceratium kepingmai	*Proceratium kepingmai*. Kunming, Yunnan Province, China	Chenyan Liao	1,2
Paraponera clavata	Portrait of a bullet ant worker. Misahuallí, Napo, Ecuador	Alex Wild	1,2,4
Stigmatomma rubiginosum	*Stigmatomma rubiginosum*. The *Stigmatomma* genus is characterized by its toothed mandibles, presumably an adaptation for capturing arthropod prey. Kunming, Yunnan Province, China	Chenyan Liao	1,2,4
Harpegnathos venator	*Harpegnathos venator*. Kunming, Yunnan Province, China	Chenyan Liao	1,2,4
Dorylus sp.	Most army ant lineages have evolved a highly flexible two-segmented waist that allows them greater use of their stinger, but *Dorylus* is unusual in that it retains the ancestral condition of a single waist segment. St. Lucia, KZN, South Africa.	Alex Wild	1,2,4
Tetraponera mocquerysi	Portrait of *Tetraponera mocquerysi*, one of the more common members of its genus across equatorial Africa. Kibale forest, Uganda	Alex Wild	1,2,4
Myrmecia nigrocincta	A worker of *Myrmecia nigrocincta*	Chengyuan Liu	1,2,4
Tapinoma melanocephalum	The Ghost Ant, *Tapinoma melanocephalum*	Chenyan Liao	1,2,4
Camponotus nicobarensis	A minor worker of the *Camponotus nicobarensis*. Kunming, Yunnan Province, China	Chenyan Liao	1,2,4
Typhlomyrmex rogenhoferi	*Typhlomyrmex* is a common but poorly understood genus of cryptobiotic ants in the new world tropics. Villa Carmen, Peru	Alex Wild	1,2,4
Cardiocondyla obscurior	A queen of *Cardiocondyla obscurior* tending to her brood. Münster, Germany	Lukas Schrader	1,2,4
Formica obscuripes	*Formica obscuripes* nestmate workers engaging in trophallaxis, or the social sharing of liquid food. Wisconsin, USA	Alex Wild	1
Solenopsis invicta	Mandible threat by a worker of *Solenopsis invicta*. Regensburg, Germany	Lukas Schrader	1

Table S2: List of the ant pictures, content description and credits. All pictures were used with permission.

GAGA id	Species Name	Karyotype (1n)	Source
GAGA-0003	*Acromyrmex lobicornis*	19	http://www.ants.ufop.br/
GAGA-0114	*Manica rubida*	22	http://www.ants.ufop.br/
GAGA-0014	*Acromyrmex echinatior*	19	http://www.ants.ufop.br/
GAGA-0024	*Lasius flavus s1*	15	http://www.ants.ufop.br/
GAGA-0026	*Myrmecocystus cf. mendax*	15	inferred from HiC
GAGA-0004	*Messor barbarus*	21	http://www.ants.ufop.br/
GAGA-0090	*Platythyrea punctata*	43	inferred from HiC
GAGA-0177	*Lasius niger*	15	http://www.ants.ufop.br/
GAGA-0187	*Plagiolepis pygmaea*	9	http://www.ants.ufop.br/
GAGA-0221	*Camponotus fellah*	20	http://www.ants.ufop.br/
GAGA-0527	*Tetramorium atratulum*	14	http://www.ants.ufop.br/
GAGA-0245	*Monomorium pharaonis*	11	http://www.ants.ufop.br/
GAGA-0084	*Aphaenogaster subterranea*	11	http://www.ants.ufop.br/
NCBI-0001	*Ooceraea biroi*	14	http://www.ants.ufop.br/
NCBI-0002	*Solenopsis invicta*	16	http://www.ants.ufop.br/
OUT-0001	*Formica selysi*	27	https://www.ncbi.nlm.nih.gov/pmc/articles/PMC9543797/

Table S3: List of species with chromosome-level assemblies and karyotype information available. Inferred = haploid karyotype inferred from HiC assemblies.
